# A Structural Landscape Depiction of Dynamic Stability Centers of Local Structure in Protein Thermostability Engineering

**DOI:** 10.34133/research.1054

**Published:** 2026-01-05

**Authors:** Xu Qiu, Huan Liu, Peizhi Song, Xiaoran Cheng, Wanjing Wu, Siyang He, Weiwei Wang, Ping Xu, Hongzhi Tang

**Affiliations:** State Key Laboratory of Microbial Metabolism and School of Life Sciences & Biotechnology, Shanghai Jiao Tong University, Shanghai 200240, P.R. China.

## Abstract

Engineering protein thermostability is a key aspect of rational protein design, aiming to broaden the applicability of enzymes and enhance their industrial utility. In this study, we introduce a strategy for identifying and reinforcing dynamic stability centers of local structure (DSCLSs) to improve protein thermostability. A DSCLS comprises key structural residues and their interactions, representing the structural basis of protein stability. Molecular dynamics, cross-correlation amino acid networks, and other analytical techniques were integrated into the method. This approach was initially inspired by thermostability engineering of exodiol dioxygenases (EDOs). The method was validated through mutational analyses of mesophilic EDO MT-2, CpKR (ketoreductase from *Candida parapsilosis*), and CaPETase (polyethylene terephthalate hydrolase from *Cryptosporangium aurantiacum*). Subsequently, we applied the approach to engineer thermostability in xp-EctC (ectoine synthase) from *Rhodococcus* and mesophilic EDO L1 from *Bacillus*, with the best-performing mutants showing *T*_m_ increases of ~15 °C. Notably, the catalytic efficiency of the optimal mesophilic EDO L1 mutant (T70Y) was 1.6-fold higher than that of the wild type at 60 °C, while the xp-EctC mutant (I2R) exhibited a 2.1-fold increase over the wild type. By characterizing and enhancing DSCLSs, this work presents a practical and generalizable strategy for thermostability engineering that also reduces mutational screening efforts, offering important potential for industrial applications.

## Introduction

Protein stability arises from a delicate equilibrium between folded and unfolded states [1,2], which can be disrupted by environmental stressors such as high temperature, organic solvents, extreme pH, and high ionic strength [3–7]. Thermostability, a specific form of protein stability, enables proteins to retain their folded conformation and function at elevated temperatures [1,8], often conferring longer half-lives and enhanced catalytic efficiency under such conditions [9,10].

Thermostable proteins can be sourced from thermophilic organisms [5,11,12] or generated through protein engineering [13–15]. Among the various strategies for thermostability engineering, identifying and enhancing structural features that confer thermal resistance is crucial [16–18], although these features often vary widely across proteins [19–21]. Rational design approaches, such as restoring conserved residues [12,22,23], reconstructing ancestral sequences [10,24], introducing specific point mutations [15,25], artificially forming new noncovalent interactions [14,22], and applying machine learning to analyze sequence features [13,17,26], have all demonstrated success in improving protein thermostability and activity. However, many of these methods lack a holistic, structure-based perspective for identifying key stabilizing residues [17]. These residues may act as allosteric regulators, exerting a broader influence over protein function and stability. Moreover, such approaches often involve laborious mutation screening with low hit rates [17,27].

Treating protein structure as a network of noncovalent interactions has become a valuable framework for thermostability engineering [28–30]. Molecular dynamics (MD) simulations [22,31,32] are commonly used to track conformational changes [33,34], while cross-correlation amino acid networks (CCAANs) [27,33,35] and community analyses [36,37] help reveal spatial and dynamical interdependencies among residues [29,34,38–40].

In this study, we investigated 3 thermophilic exodiol dioxygenases (EDOs) (1012, 1028, and 1371) from *Hydrogenibacillus* sp. N12 and performed a comprehensive structural analysis of the EDO family, key phenyl-ring-cleaving enzymes involved in polycyclic aromatic hydrocarbon degradation [11,41]. We identified critical thermostability-related features of EDOs and demonstrated that a rationally designed triple mutant (57F/107F/229F) of the mesophilic EDO MT-2 increased *T*_m_ by approximately 25 °C and improved catalytic efficiency by 1.23-fold at 70 °C.

Based on these data and further analyses, we developed a thermostability engineering method centered on identifying and enhancing dynamic stability centers of local structure (DSCLSs), analogous to protein sectors, which are coevolving residue networks that underlie biochemical function [42–44]. This approach allows the recognition of stable local structural regions that contribute to global thermostability. Application of this method successfully identified DSCLSs in mesophilic EDO MT-2, CpKR (ketoreductase from *Candida parapsilosis*) [45], and CaPETase (polyethylene terephthalate hydrolase from *Cryptosporangium aurantiacum*) [22], with enhanced or newly formed DSCLSs observed in their optimal mutants. We then extended the approach to guide mutations in mesophilic EDO L1 from *Bacillus* and xp-EctC [46] from *Rhodococcus*, resulting in approximately 15 °C increases in *T*_m_. The mesophilic EDO L1 mutant T70Y showed a 1.6-fold increase in catalytic efficiency at 60 °C, while the xp-EctC I2R mutant exhibited a 2.1-fold improvement over the wild type (WT).

## Results

### Characterization of different EDOs

**Fig. 1.**
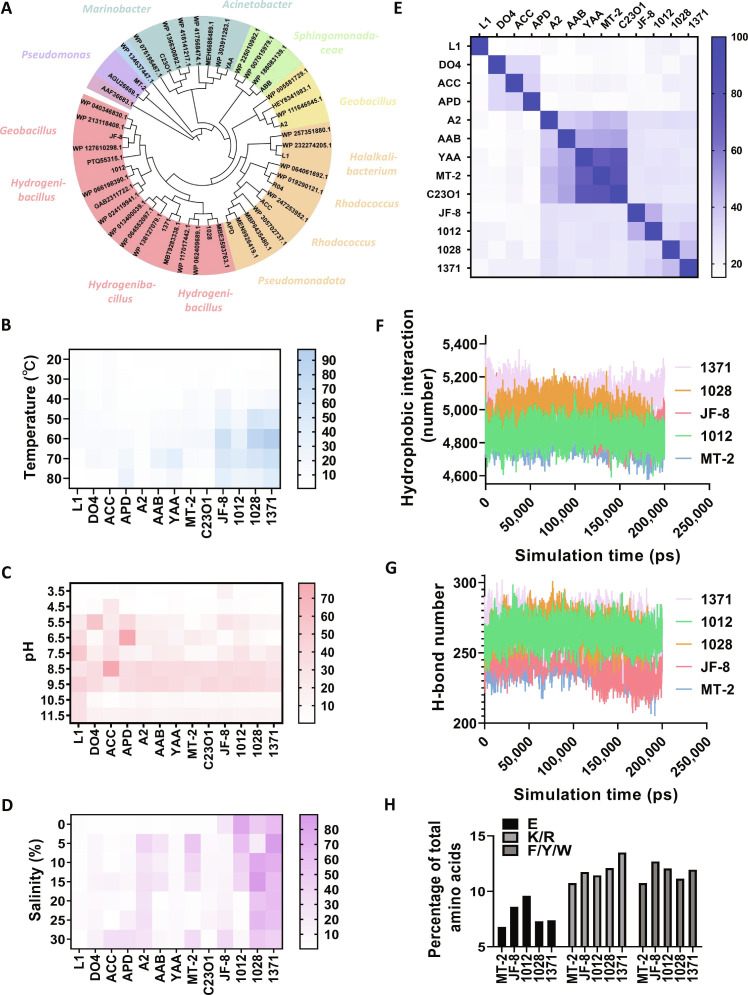
Characterization of diverse exodiol dioxygenases (EDOs). (A) Maximum likelihood phylogenetic tree of thermophilic EDOs 1012, 1028, and 1371 from *Hydrogenibacillus* sp. N12 alongside 10 reported EDOs and 37 UniProt-recorded EDOs. Colored regions denote distinct phylogenetic clusters. The species origins of 13 selected EDOs are annotated along the tree. (B to D) Relative catalytic efficiency assays of selected EDOs under varying temperature, pH, and salinity conditions. Colored bars indicate relative catalytic efficiency. A darker color represents a higher catalytic efficiency. Reactions were performed in triplicate; data are presented as mean ± SD. (E) Percent similarity matrix of protein sequences among the 13 selected EDOs. Color gradients represent sequence similarity; exact values are provided in Table S1. (F) Hydrophobic interaction profiles of mesophilic EDO MT-2 and thermophilic EDOs JF-8, 1012, 1028, and 1371, obtained via molecular dynamics (MD) simulation. The quantity of hydrophobic interaction profiles varies with simulation time, and its change reflects changes in the structure’s stability. Each EDO is depicted as a uniquely colored curve. (G) H-bond interaction analysis of the same EDOs as in (F), also via MD simulation, with distinct colors representing different enzymes. (H) Relative abundance of selected amino acid residues (lysine [K], arginine [R], glutamic acid [E], phenylalanine [F], tyrosine [Y], and tryptophan [W]) in mesophilic EDO MT-2 and thermophilic EDOs JF-8, 1012, 1028, and 1371, shown as percentage of total amino acids.

EDOs are positioned at critical junctions in bacterial degradation pathways of aromatic compounds and serve as rate-limiting enzymes in catalytic oxidative benzene cracking. To elucidate their structural and functional diversity within a phylogenetic context, 13 representative EDOs were selected through comprehensive literature mining and sequence alignment, including 3 thermophilic enzymes (1012, 1028, and 1371) from *Hydrogenibacillus* sp. N12 [11,41]. Phylogenetic analysis grouped these enzymes into 8 main clusters, with 1012, 1028, 1371, and thermophilic EDO JF-8 forming a single cluster, indicative of shared thermostable features (Fig. 1A).

The 3 thermophilic EDOs from *Hydrogenibacillus* sp. N12 were successfully purified (Fig. S1), and structural comparison revealed high similarity among them (Fig. S2), with root mean square deviations (RMSDs) of 1.065 Å (1028 to 1012) and 1.371 Å (1371 to 1012). Thermophilic EDO 1371 displayed the highest catalytic efficiency at 60 °C, while EDOs 1012 and 1028 were most active at 70 °C. Remarkably, all 3 EDOs retained catalytic activity at 90 °C (Fig. S3). Circular dichroism spectroscopy showed no obvious changes in secondary structure from 30 to 90 °C in the 200- to 210-nm range (Fig. S4). These findings laid the groundwork for investigating EDO thermostability and developing rational engineering strategies.

Next, we assessed the environmental adaptability of all 13 purified EDOs (Fig. S5) under varying temperature, pH, and salinity conditions (Fig. 1B to D). Thermophilic EDOs 1371, 1028, 1012, and JF-8 demonstrated notable halotolerance, with thermophilic EDO 1012 being especially robust (Fig. 1D). Several mesophilic EDOs also exhibited strong resistance to extreme environments, including hyperalkaline conditions (e.g., mesophilic EDO L1) and high salinity (e.g., mesophilic EDOs A2 and MT-2) (Fig. 1C and D). Among these, mesophilic EDO MT-2 from *Pseudomonas putida*—the earliest crystallized and most structurally characterized EDO—displayed the broadest sequence similarity across the 13 selected enzymes (Fig. 1E and Table S1). Based on their diverse thermostability and environmental profiles, mesophilic EDO MT-2 and thermophilic EDOs JF-8, 1012, 1028, and 1371 were selected for further structural analysis and thermostability engineering.

All EDOs share a conserved architecture composed of 4 external α-helices and 2 internal β-sheet hydrophobic domains, as exemplified by mesophilic EDO MT-2 (Figs. S6 and S7). MD simulations were employed to analyze conformational flexibility in mesophilic EDO MT-2 and thermophilic EDOs JF-8, 1012, 1028, and 1371 [22]. Thermophilic EDOs exhibited more extensive hydrophobic interactions (notably in 1371, 1028, and JF-8) and more hydrogen bonds (especially in 1371, 1012, and 1028), reflecting a denser network of hydrophobic stacking and interdomain connections (Fig. 1F and G). Sequence analysis of the 4 thermophilic EDOs (Table S2) revealed an enrichment of positively charged (lysine [K] and arginine [R]), negatively charged (glutamic acid [E]), and aromatic residues (phenylalanine [F], tyrosine [Y], and tryptophan [W]) that contribute to hydrophobic interactions and hydrogen bonding (Fig. 1H). For instance, highly connected residues clustered around the α1 region were predominantly aromatic or charged (Fig. S8).

In contrast, mesophilic EDO MT-2 exhibited lower solvent accessibility and a smaller radius of gyration relative to those of the thermophilic EDOs (Figs. S9 and S10), yet classical thermophilic structural traits were less pronounced in the 4 thermophilic EDOs. Similar patterns were observed in backbone fluctuations (Fig. S11) and surface electrostatic properties (Fig. S12), suggesting compensatory mechanisms between conformational dynamics and thermostability [32,44].

These results support the notion that internal compactness—achieved through strengthened hydrophobic stacking and enhanced interdomain connectivity—is a key determinant of protein thermostability [10,28]. The distribution and density of stabilizing residues play a critical role in maintaining these compact structural frameworks under thermal stress [47].

### Protein thermostability engineering of mesophilic EDO MT-2

**Fig. 2.**
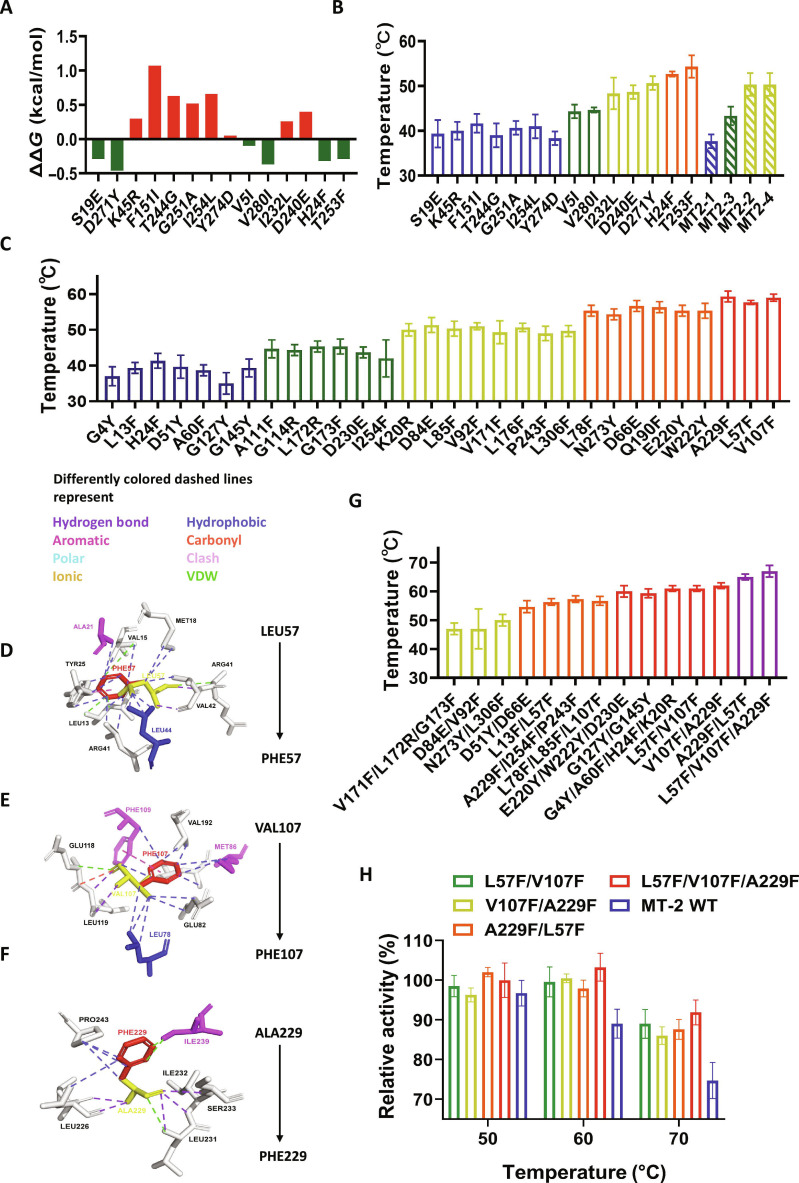
Structural mutation analysis at conserved sites for enhancing thermostability. (A) Predicted changes in folding free energy (ΔΔ*G*) of conserved-site mutations in mesophilic EDO MT-2 calculated by FoldX. Negative numbers indicate structural strengthening, while positive numbers indicate structural weakening. (B and C) Melting temperatures (*T*_m_) of individual conserved-site mutants and structure-guided mutants in mesophilic EDO MT-2. Color codes indicate different temperature tolerance categories. Reactions were performed in triplicate; data are presented as mean ± SD. (D to F) Alterations in noncovalent bonding patterns caused by mutations L57F, V107F, and A229F. The mutated amino acid forms more noncovalent bonds around. The local structure in mutations is stabilized by hydrogen bonds and aromatic interactions. In L57F, increased noncovalent bonds appear among PHE57:VAL5/ALA21:TYR25/LEU13:ARG41. In V107F, increased noncovalent bonds appear among PHE107:PHE109/VAL192:MET86. In A229F, increased noncovalent bonds appear among ILE239:PRO243. Central residues are shown as sticks; surrounding interacting residues before mutation are colored blue, and those after mutation are purple. Noncovalent bonds are shown as dashed lines in various colors. (G) Melting temperatures (*T*_m_) of combined mutants in mesophilic EDO MT-2. Color codes indicate different temperature tolerance categories. Reactions were performed in triplicate; data are presented as mean ± SD. (H) Catalytic efficiency of wild-type (WT) mesophilic EDO MT-2 and the optimal combinatorial mutant L57F/V107F/A229F across different temperatures. Reactions were performed in triplicate; data are presented as mean ± SD.

Phylogenetically conserved residues are often functionally and structurally crucial, making them ideal targets in protein engineering to enhance catalytic efficiency and thermostability [12,22]. Conserved sites identified in the 4 thermophilic EDOs were mapped and applied to the mesophilic EDO MT-2 (Figs. S13 and S14). FoldX was used to predict changes in folding free energy upon mutation, and substitutions at positions V5I, S19E, H24F, T253F, D271Y, and V280I were predicted to improve protein stability (Fig. 2A).

Thermal shift assays (TSAs) confirmed increased thermostability for several mutations, including V5I, H24F, I232L, D240E, T253F, and D271Y (Fig. 2B and Fig. S15), although some discrepancies were observed compared with FoldX predictions. These differences may reflect compensatory or antagonistic effects from global structural changes that offset local stabilizing effects [12]. Among these, H24F and T253F increased *T*_m_ to approximately 55 °C, while I232L, D240E, and D271Y raised it to around 50 °C (Fig. 2B). The H24F and T253F mutations enhanced π–π interactions and hydrogen bonding density, I232L optimized hydrophobic packing, and D240E introduced new salt bridges (Fig. S16). However, none of the tested combinations of conserved-site mutations exceeded a *T*_m_ of 55 °C, likely due to epistatic effects that limited additive thermostability.

To address these limitations, we implemented a structure-guided mutation strategy (Fig. S17), selecting 30 candidate residues in mesophilic EDO MT-2 for mutagenesis based on local structural context (Figs. S15 and S18 and Table S3). TSA revealed 17 mutations that significantly improved thermostability, with the most effective—A229F (α3), L57F (β4), and V107F (β6)—raising *T*_m_ to approximately 60 °C (Fig. 2C). L57F and V107F mutations markedly strengthened local noncovalent interaction networks (Fig. 2D and E), while A229F enhanced interactions with I239, reinforcing α3 connectivity (Fig. 2F).

To assess how specific structural regions contribute to overall thermostability, combinations of the 30 mutations were tested according to amino acid communities (Fig. S19). Notably, the triple mutant L57F/V107F/A229F and the double mutant A229F/L57F both raised *T*_m_ to ~65 °C (Fig. 2G). Other combinations—L57F/V107F, V107F/A229F, G4Y/A60Y/H24F/K20R (α1), and E220Y/W222Y/D230E (α3)—increased *T*_m_ to ~60 °C (Fig. 2G). Importantly, the L57F/V107F/A229F mutant retained ~90% of its maximal catalytic activity at 70 °C, representing a 1.23-fold increase over that of the WT (Fig. 2H). The relatively modest *T*_m_ gain in this mutant, despite its enhanced stability, may be attributed to structural epistasis limiting the additive effects of the combined mutations [31,48–50].

Experimental data suggest that internal hydrophobic domains and interdomain connectivity are central to the thermostability of EDOs. However, both conserved-site-based and structure-guided mutagenesis approaches face challenges—chiefly, high screening workloads and low hit rates. These limitations likely stem from the dynamic nature of domain interactions within the protein, which complicates the accurate prediction and identification of effective stabilizing mutations.

### Establishment and validation of the DSCLS engineering strategy

The 3-dimensional architecture of proteins is governed by the amino acid network (AAN), in which steric hindrance and peptide chain interactions determine the overall structure’s compactness and stability [27,47]. Optimizing the topological organization of this network is a proven strategy for enhancing protein thermostability [39,48]. Within the AAN, strong hubs are typically composed of aromatic or charged residues, whereas weak hubs often include leucine or isoleucine [28,39]. Accurately distinguishing between strong and weak hubs is critical for selecting optimal mutation sites and directions during protein engineering.

**Fig. 3.**
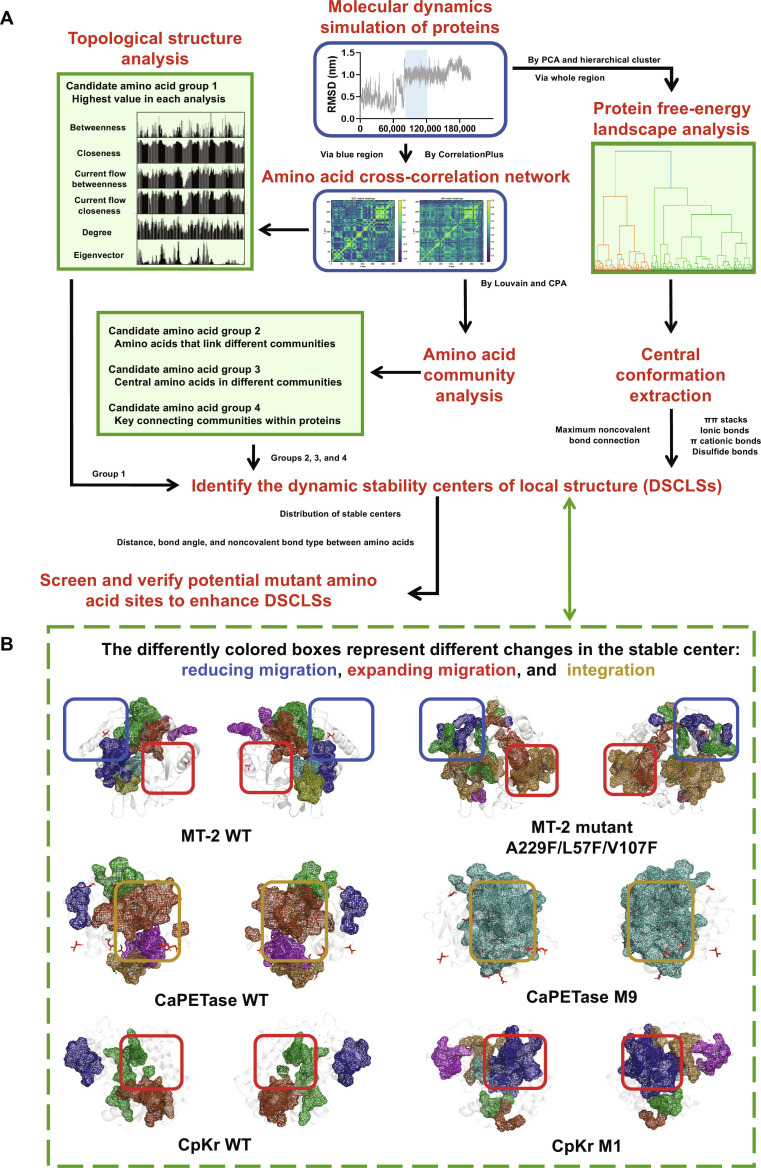
Development and validation of the dynamic stability center of local structure (DSCLS) engineering strategy. (A) Workflow for identifying DSCLSs and selecting mutation sites. The entire set of root mean square deviation (RMSD) values was extracted from GROMACS-based MD simulations and used for protein free-energy conformational clustering. Stable regions of RMSD (blue regions) were input into cross-correlation amino acid network (CCAAN) analysis using CorrelationPlus. The identification of key amino acids in protein structures can be facilitated by analyzing the topological structure and community of amino acid networks. These amino acids have been demonstrated to play pivotal roles in facilitating connection and communication. Subsequently, the representative conformations of these key amino acid sites are displayed in relation to the overall structural changes of the protein, which represent DSCLSs. (B) DSCLS distributions in WT and mutant forms of mesophilic EDO MT-2 (A229F/L57F/V107F), polyethylene terephthalate (PET) hydrolase (CaPETase^WT^ and CaPETase^M9^: R242C, S291C, R198K, G196T, L180C, A202C, V129T, A155R, and N109A), and ketoreductase (CpKR^WT^ and CpKR^M1^: V24L, V118I, L177F, and T181A). The coverage area of DSCLSs is indicative of the structural basis for protein stability enhancement. The alteration in the coverage area of DSCLSs indicates a transition in protein structural strength. For each protein, the 2 conformational states are shown as mirror images. Actual mutation sites are marked with red sticks. DSCLS regions are highlighted in various colors; different boxed regions indicate distinct DSCLS alterations. PCA, principal component analysis; CPA, clique percolation algorithm; DCC, dynamic cross-correlation; LMI, linear mutual information.

To systematically improve protein thermostability, we developed an integrated engineering approach (Fig. 3A) centered on identifying DSCLSs. The primary objective of this method is to identify and analyze amino acids, which have been demonstrated to play a pivotal role in the interconnection and integration of different protein structural regions within the AAN. These amino acids, in conjunction with their surrounding counterparts (DSCLSs), collectively contribute to the protein’s overall stability. This method combines MD simulations [22], CCAAN analysis [27,51], topological structure profiling [51], amino acid community clustering [27,51], and free-energy landscape (FEL) mapping [16]. DSCLSs are defined as topologically strong hubs in the AAN, comprising key structural residues and their stabilizing noncovalent interactions.

Maximal noncovalent interaction networks were constructed by extracting representative conformations from the FEL, derived from MD simulations [22]. To evaluate correlated spatial movement among residues, 2 independent CCAAN metrics were applied: normalized dynamic cross-correlation (NDCC) and normalized linear mutual information (NLMI) [27,51]. Key structural residues were then identified through centrality-based topological analysis and amino acid community mapping. These residues, classified as high-centrality [51], linking [39], or community-bridging nodes [43], were isolated from catalytic pockets and weakly connected regions to define DSCLS subnetworks [52].

Compared to mesophilic EDO MT-2 WT (Fig. 3B and Fig. S20), the triple mutant A229F/L57F/V107F displayed fused and relocated DSCLSs (Fig. 3B and Fig. S21). This fusion significantly expanded the spatial influence of DSCLSs, correlating with enhanced thermostability. A similar expansion of dominant DSCLSs occupying the majority of the protein’s structure was observed in the thermophilic EDO 1012 (Figs. S22 and S23).

To validate the method’s generalizability, we analyzed previously engineered proteins, including the polyethylene terephthalate (PET) hydrolase CaPETase [22] and the ketoreductase CpKR [45]. CaPETase^WT^ exhibited a *T*_m_ of ~66.8 °C, while the optimized mutant CaPETase^M9^ reached ~83.5 °C, with a 41.7-fold increase in catalytic efficiency at 60 °C. Similarly, CpKR^WT^ had a *T*_m_ of ~74.9 °C, whereas the mutant CpKR^M1^ (V24L, V118I, L177F, and T181A) improved thermostability by ~10 °C.

In CaPETase^M9^ (R242C, S291C, R198K, G196T, L180C, A202C, V129T, A155R, and N109A), DSCLS analysis revealed a single fused hub (Fig. 3B and Figs. S24 and S25), contrasting with the multiple discrete DSCLSs in CaPETase^WT^. Key stabilizing interactions were attributed to R242C/S291C and L180C/A202C, which reinforced flexible loop regions, while R198K extended DSCLS influence into the α-helix domain. In CpKR^M1^ (V24L, V118I, L177F, and T181A), 4 mutations reduced steric hindrance and facilitated tighter hydrophobic domain packing (Fig. 3B and Figs. S26 and S27), which allowed 2 previously separated local DSCLSs to merge, improving overall thermostability.

### Application of the DSCLS engineering strategy

We applied the DSCLS-based strategy to engineer thermostability in 2 target enzymes: mesophilic EDO L1 from *Bacillus* and ectoine synthase xp-EctC [46] from *Rhodococcus*. Mutation site selection was guided by their potential to form or strengthen noncovalent interactions with existing DSCLSs or to nucleate new DSCLS hubs. Design direction focused on promoting denser or stronger interdomain connectivity, particularly involving charged and aromatic residues.

**Fig. 4.**
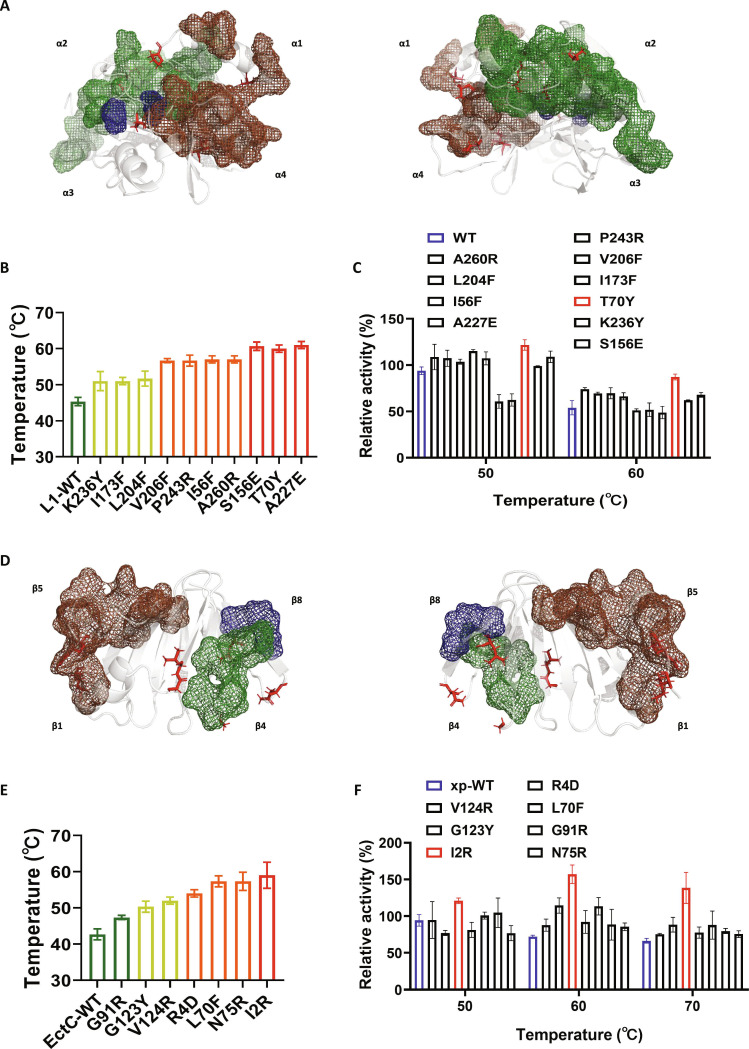
Application of the DSCLS-guided engineering strategy. (A) DSCLS distribution in WT mesophilic EDO L1. Mirror-symmetric conformations are shown for each protein. Mutation sites are highlighted in red sticks; DSCLS regions are color coded, and boxes denote distinct conformational changes. (B) *T*_m_ values of mutations introduced in mesophilic EDO L1. Colors represent different thermostability ranges. Reactions were performed in triplicate; data are presented as mean ± SD. (C) Catalytic efficiency of WT and mutant forms of mesophilic EDO L1 under different temperature conditions. Reactions were performed in triplicate; data are presented as mean ± SD. (D) DSCLS distribution in WT ectoine synthase xp-EctC. (E) *T*_m_ values of mutations introduced in xp-EctC. (F) Catalytic efficiency of the WT and mutant forms of xp-EctC under different temperature conditions.

In mesophilic EDO L1, only 3 major DSCLSs were initially identified (Fig. 4A and Fig. S28). Drawing from insights in mesophilic EDO MT-2, we introduced 10 mutations—K236Y, I173F, L204F, V206F, P243R, I56F, A260R, S156E, T70Y, and A227E—to expand DSCLS influence (Fig. S29 and Table S4). These mutations targeted the enhancement of internal hydrophobic packing and interdomain linkages. Structural analysis confirmed the establishment of the intended noncovalent interactions and revealed a reduction in local rigidity, particularly due to S156E, I173F, A227E, and P243R (Fig. S30). TSA results showed that all 10 mutations conferred thermostability improvements, with S156E, T70Y, and A227E increasing *T*_m_ to ~60 °C (Fig. 4B). Catalytic assays further confirmed improved performance across all variants at 60 °C (Fig. 4C).

The β-rich xp-EctC enzyme from *Rhodococcus*, composed of 136 residues, has limited internal space dominated by its catalytic pocket. Two surface-localized DSCLSs were identified (Fig. 4D and Fig. S31). In contrast, the thermophilic ectoine synthase (*Hydrogenibacillus* sp. N12) exhibited 5 DSCLSs distributed across surface loops and β-regions (Figs. S32 and S33). Based on this, 7 mutations—G91R, G123Y, V124R, R4D, L70F, N75R, and I2R—were designed to form new DSCLS hubs via strengthened loop–β-sheet interactions (Fig. S34 and Table S5). Structural analysis confirmed enhanced noncovalent linkages and decreased local rigidity at several sites (I2R, R4D, L70F, G91R, and G123Y; Fig. S35). TSA confirmed the thermostabilizing effect of all 7 mutations: I2R raised *T*_m_ to ~60 °C, while R4D, L70F, and N75R reached ~55 °C (Fig. 4E). Catalytic efficiency measurements validated functional improvements across all variants at 60 and 70 °C (Fig. 4F).

Based on the identification of DSCLSs, we pinpointed mutation targets for engineering. Enhancing, expanding, or introducing new DSCLSs through rational mutagenesis significantly improved protein thermostability. Results from all engineered proteins further underscored the importance of random combinatorial testing of mutation sites, once optimal single-site mutations were identified, to achieve the best thermostability enhancement [48].

## Discussion

Engineering protein thermostability is crucial for both industrial applications and laboratory research, facilitated by rational design, computational tools, and other strategies [24]. Despite progress in thermostability engineering, the efficient identification of beneficial mutation sites remains a major bottleneck, largely due to a limited understanding of the structural features governing protein stability.

In this study, we introduced 3 thermophilic EDOs—1012, 1028, and 1371—from *Hydrogenibacillus* sp. N12, all of which retained catalytic activity at 90 °C. These thermophilic EDOs represent key degradation components in synthetic biology applications. Guided by the structural thermostability features of thermophilic EDOs 1012, 1028, 1371, and JF-8, we introduced conserved and structure-informed mutations into the mesophilic EDO MT-2. The best-performing triple mutant (57F/107F/229F) showed an approximate 25 °C increase in *T*_m_ and a 1.23-fold increase in catalytic efficiency over the WT at 70 °C. These findings suggest that internal hydrophobic domains and interdomain interactions are critical contributors to the thermostability of EDOs. Nonetheless, the labor-intensive screening process and low rate of positive hits in mesophilic EDO MT-2 highlight the challenges of mutation site selection, likely due to dynamic interdomain relationships during protein fluctuations.

During thermal fluctuations, proteins behave as dynamic amino acid interaction networks, where a complex architecture of strong and weak interaction hubs connects different domains across diverse spatial scales [27,35,47]. In such networks—commonly referred to as AANs or CCAANs—allosteric communication contributes to global stability [28,37,38], although only a few subnetworks extend into distal regions [38,48]. Building on this framework, we proposed a novel thermostability engineering strategy centered on the identification and enhancement of DSCLSs. A DSCLS functions as a strong interaction hub within an AAN and is composed of key structural residues and their interactions, representing a topological transformation landscape. DSCLS hubs represent dynamic, locally confined stability centers that shape the global topological structure of a protein. Mutations—whether through substitution [1,32], insertion [53–55], or deletion [54]—can induce conformational changes such as main-chain shifts [32,56], α-helix bending [55,56], and reduction in internal hydrophobic cavities [20,37,56]. These structural rearrangements influence the formation, expansion, relocation, and recombination of DSCLSs to achieve misfolding avoidance [57,58], which nevertheless persist as dynamic stabilizing cores within local domains. By targeting original amino acid positions for either reinforcing existing DSCLSs or creating new ones, this approach can efficaciously enhance thermostability while minimizing deleterious mutations. In contrast, randomly selected mutations generally fail to impact protein structure or function unless they occur at critical network hubs.

We validated this strategy across several reported proteins. For mesophilic EDO MT-2 from *P. putida*, CpKR (ketoreductase), and CaPETase (PET hydrolase), DSCLS-guided description explained the reason for thermostable variants. The approach was subsequently applied to engineer mesophilic EDO L1 from *Bacillus* and xp-EctC (ectoine synthase) from *Rhodococcus*. In both cases, optimal mutations resulted in ~15 °C increases in *T*_m_. Notably, the T70Y mutation in mesophilic EDO L1 improved catalytic efficiency 1.6-fold over that of the WT at 60 °C, and the I2R mutation in xp-EctC yielded a 2.1-fold enhancement relative to that of the WT.

In summary, this study presents a dynamic, network-based strategy for protein thermostability engineering by identifying and enhancing DSCLSs. Other engineering methods for improving the thermostability of proteins come with certain issues. Numerical estimates of free energy are often inaccurate. Furthermore, the artificial design of the internal connections or the surface electrostatic potential energy of proteins can yield adverse outcomes, including directly reducing enzyme catalytic activity. The engineering of protein thermostability or enzyme activity through artificial intelligence has been shown to yield momentous benefits. Nevertheless, the repeated adjustment of parameters and the training of test sets are extremely complex, and the direction of engineering may be highly specific. This approach in our study provides a precise and efficient method for enhancing biocatalysts in industrial settings, substantially reducing the mutational screening workload due to its high success rate.

## Materials and Methods

### Plasmid construction and protein purification

Genes encoding the 3 predicted EDOs were amplified from the genomic DNA of *Hydrogenibacillus* sp. N12 and cloned into the pET28a expression vector. Additional genes were inserted into pET28a via gene synthesis. All constructs were transformed individually into *Escherichia coli* BL21(DE3). Cultures were grown in Luria–Bertani medium at 37 °C until reaching an OD_600_ of 0.6 to 0.8, at which point isopropyl β-d-1-thiogalactopyranoside was added to a final concentration of 0.2 mM. Cells were incubated at 16 °C for 12 to 16 h and then harvested by centrifugation (6,000 rpm, 10 min). Pellets were resuspended in buffer A (25 mM Tris–HCl, 300 mM NaCl, and 20 mM imidazole) and lysed using a high-pressure homogenizer (900 bar, 50 ml/min for 2 min). Lysates were cleared by centrifugation (10,000 rpm, 60 min, 4 °C). Proteins were purified via Ni-NTA affinity chromatography and eluted using imidazole gradient buffers. Final protein preparations were stored in buffer B (15 mM Tris–HCl and 150 mM NaCl). Protein purity was confirmed by sodium dodecyl sulfate–polyacrylamide gel electrophoresis following mixing with 5× sodium dodecyl sulfate loading buffer.

### Verification of point mutation

Site-directed mutants were generated using the pET28a plasmid, which contains the target gene, and primers encoding the desired mutations with a 20- to 40-bp overlap. Polymerase chain reaction products (20 μl) were treated with 1 μl of *Dpn*I and 1 μl of CutSmart buffer at 37 °C for 4 h to degrade template DNA. The reaction mixtures were transformed into *E. coli* DH5α and sequenced to confirm successful mutagenesis. Verified plasmids were then transformed into *E. coli* BL21(DE3) for protein expression and purification as described above.

### Enzyme catalytic efficiency assays

Enzymatic activity was assessed in 700-μl reaction mixtures containing 25 mM Tris–HCl (pH 7.5) and 40 μM catechol. Reactions were preincubated at the target temperature for 5 min before initiating the reaction by adding 10 μM protein. After 3 min of incubation, reactions were quenched with 5 μl of 5 M HCl. Product formation was monitored at 375 nm using a UV-2550 spectrophotometer equipped with a temperature-controlled chamber (ectoine reactions were monitored at 215 nm). All assays were conducted in triplicate, and results are presented as mean ± SD.

### Thermal shift assay

TSA was used to evaluate protein thermostability. Upon heating, protein unfolding exposes hydrophobic residues that bind to the fluorescent dye Sypro Orange, enabling detection via fluorescence. Reactions (20 μl) contained 10 μl of 2× Sypro Orange, 10 μl of 20 mM Tris–HCl (pH 7.5), and 5 to 10 μg of purified protein. The assay was performed in a real-time quantitative polymerase chain reaction system under the following conditions: 5 min at 20 °C, followed by 5 min at 30 °C and then 1 °C increments with 1-min holds from 30 to 95 °C. Fluorescence was recorded throughout. Each sample was measured in triplicate, and data are shown as mean ± SD.

### MD simulation

Protein structures were either predicted using AlphaFold2 or obtained from the Research Collaboratory for Structural Bioinformatics Protein Data Bank (RCSB PDB). Solvent molecules, ions, and unrelated entities were removed in PyMOL, retaining only the protein monomer. Simulations were conducted using GROMACS. Systems were solvated with TIP3P water and electrically neutralized with Na^+^ or Cl^−^ ions. Energy minimization proceeded in 2 phases: NVT and NPT equilibration, each lasting 50 ps. Systems were then heated from 0 to 300.15 K (or 333.15 K) over 50 ps and simulated for 150 ns. RMSD and interatomic distances were analyzed using Cpptraj.

### Protein FEL and stable conformation clustering

Analysis of MD trajectories (XTC format) and initial protein structures (PDB format) was used to construct FELs. A covariance matrix based on Cα atom fluctuations was computed for principal component analysis. The resulting principal component analysis data were projected into 2 dimensions, and protein conformations were classified via hierarchical clustering (predefined cluster number). The centroid of each cluster was calculated, and the representative structure nearest to each centroid was selected for further analysis.

### Protein amino acid cross-correlation network analysis

To identify key residues and inter-residue dynamics, pairwise correlation analyses were performed using XTC trajectories and initial PDB structures. The CorrelationPlus package was used to compute NDCCs and NLMI. Topological properties such as degree, closeness, betweenness, current flow closeness, current flow betweenness, and eigenvector centrality were calculated to assess each residue’s network importance.

### Amino acid community detection from cross-correlation networks

Community detection was performed based on betweenness centrality derived from NDCC and NLMI matrices. The initial community partitioning used the Louvain algorithm, which groups densely connected residues into local communities. Communities were then refined using the clique percolation algorithm. In this refinement, unvisited nodes were merged with adjacent communities based on shared *k* − 1 or *k* − 2 neighbor nodes. Community roles—leaders, followers, and key connectors—were defined based on intra- and inter-community interactions. Leaders and followers form stable intra-community clusters, while key connectors facilitate communication between communities.

## Data Availability

All data are available in the main text or the Supplementary Materials.

## Supplementary Materials

Supplementary 1 The amino acid sequences of selected proteins in the paper Figs. S1 to S35 Tables S1 to S7
