# Supplementary material for: A Structural Landscape Depiction of Dynamic Stability Centers of Local Structure in Protein Thermostability Engineering
Source: Research (Wash D C). 2026 Jan 5;9:1054. doi: 10.34133/research.1054 (PMC12766707; doi:10.34133/research.1054)
Supplement: Supplementary 1 — The amino acid sequences of selected proteins in the paper Figs. S1 to S35 Tables S1 to S7 [file research.1054.f1.zip › new-Supplementary.pdf]

A Structural Landscape Depiction of DSCLSs in Protein Thermostability Engineering

Xu Qiu[1], Huan Liu[1], Peizhi Song[1], Xiaoran Cheng[1], Wanjing Wu[1], Siyang He[1], Weiwei Wang[1]*, Ping Xu[1], and Hongzhi Tang[1]*

1. State Key Laboratory of Microbial Metabolism, and School of Life Sciences & Biotechnology, Shanghai Jiao Tong University, Shanghai, 200240, P.R. China.

***Corresponding author: H. Z. Tang or W. Wang**

Mailing address:

School of Life Sciences & Biotechnology, Shanghai Jiao Tong University, Shanghai 200240, P. R. China

E-mail:

tanghongzhi@sjtu.edu.cn (Hongzhi Tang)

oudigouzai@sjtu.edu.cn (Weiwei Wang)

Tel: +86-21-3420406

Supplementary Materials

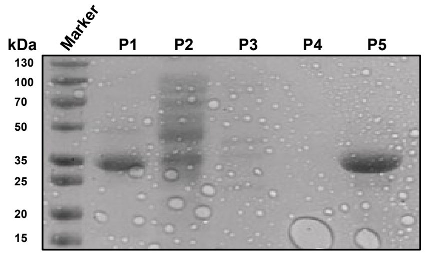

Extradiol dioxygenase 1012

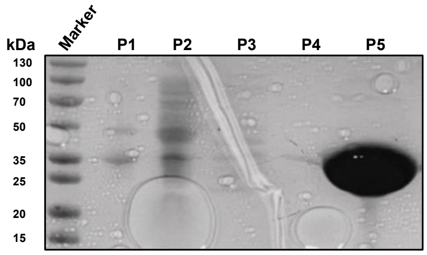

Extradiol dioxygenase 1028

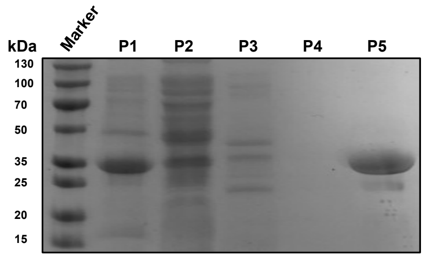

Extradiol dioxygenase 1371

Fig. S1. Protein purification of thermophilic EDOs 1012, 1028, and 1371 from *Hydrogenibacillus* N12. P1: supernatant of the cell lysate, P2: precipitation of the cell lysate, P3: flow-through solution, P4: wash solution, P5: elution solution. The purified protein is in P5 and has an apparent molecular weight of about 35 kDa.

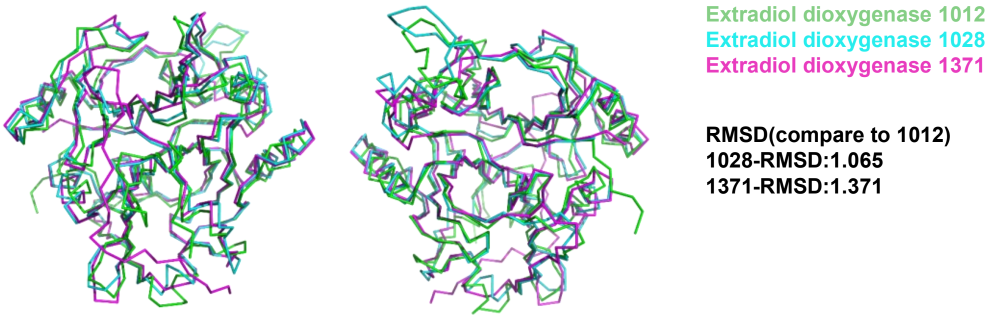

Fig. S2. Protein structure superposition of thermophilic EDOs 1012, 1028, and 1371 from *Hydrogenibacillus* N12. The two conformations of one protein are mirror images. Thermophilic EDOs 1012, 1028, and 1371 are represented by different colors.

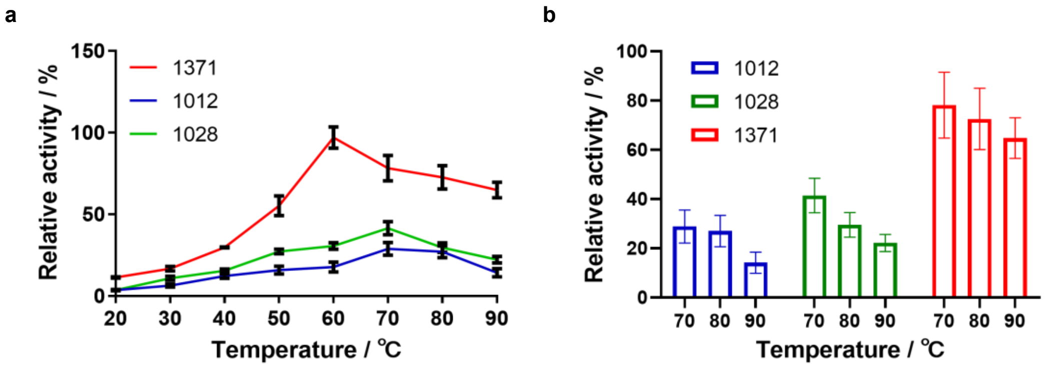

Fig. S3. Catalytic efficiency experiment of thermophilic EDOs 1012, 1028, 1371.
(a) Catalytic efficiency experiment of thermophilic EDOs 1012, 1028, 1371 from 20°C to 90°C. Thermophilic EDOs 1012, 1028, and 1371 are indicated by curves in different colors. (b) Catalytic efficiency experiment of thermophilic EDOs 1012, 1028, 1371 at 70°C, 80°C, and 90°C. Reactions were performed in triplicate; Data are presented as mean values ± SD.

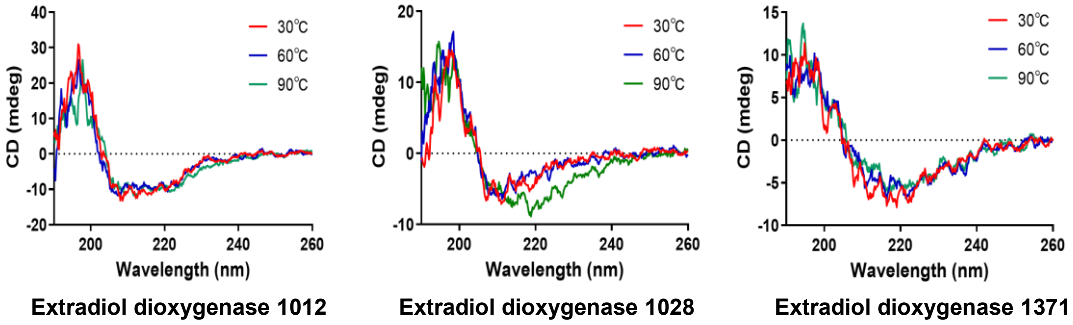

Fig. S4. Secondary structure changes of thermophilic EDOs 1012, 1028, and 1371 via Circular dichroism (CD) spectroscopy in the detection range of 190-260 nm. Thermophilic EDOs 1012, 1028, and 1371 are indicated by curves in different colors.

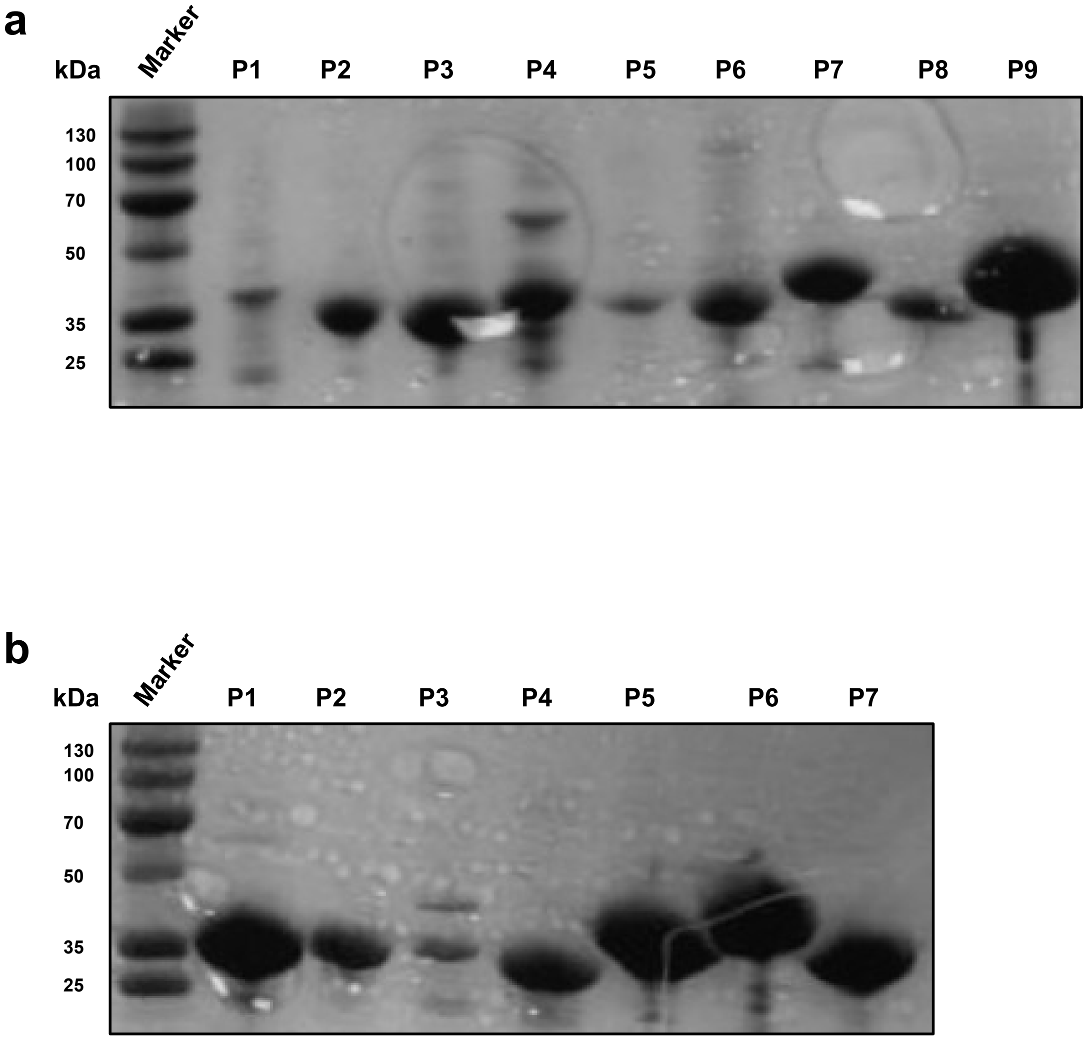

Fig. S5. Protein purification of 13 EDOs. (a) P1:C23O1-1, P2:YAA, P3:ACC, P4:JF-8, P5:1012-1, P6:ABB, P7:R04, P8:MT-2, P9:L1. **(b)** P1:APD, P2:A2, P3:1371-1 P4:C23O1-2, P5:1012-2, P6:1371-2, P7:1028. The purified proteins are about 35 kDa.

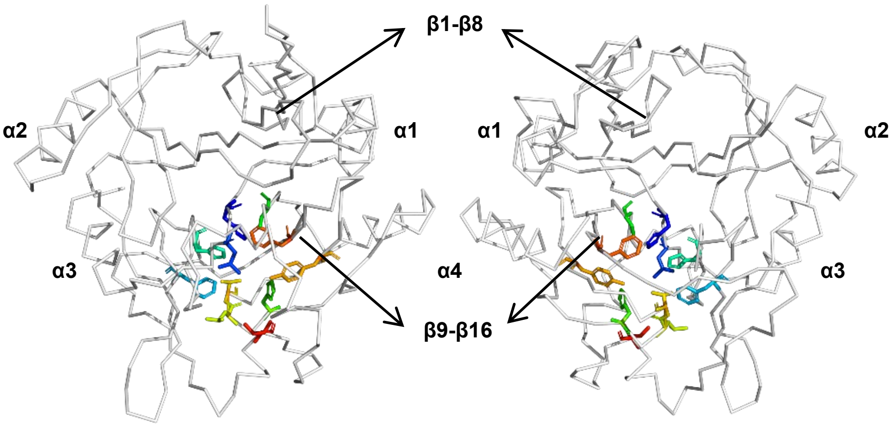

Fig. S6. The schematic structure of the mesophilic EDO MT-2. The two figures are mirror-image controls. The protein structures are presented in cartoon. The two conformations of one protein are mirror images. The colored residues represent active sites.

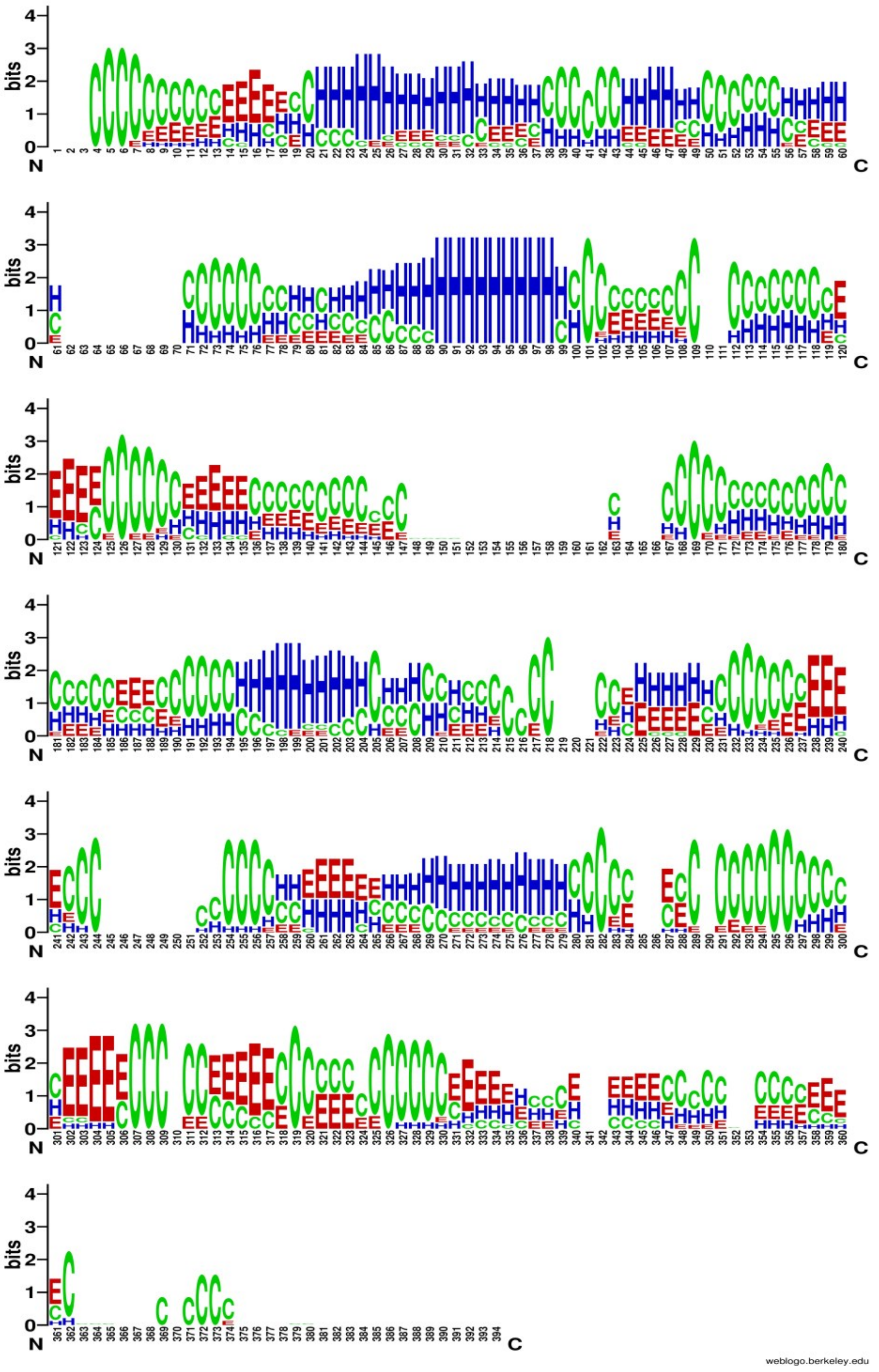

Fig. S7. Secondary structure homology of 13 EDOs. The blue area represents the α-helix regions, the red area represents the β-sheet regions, and the green area represents the loop regions.

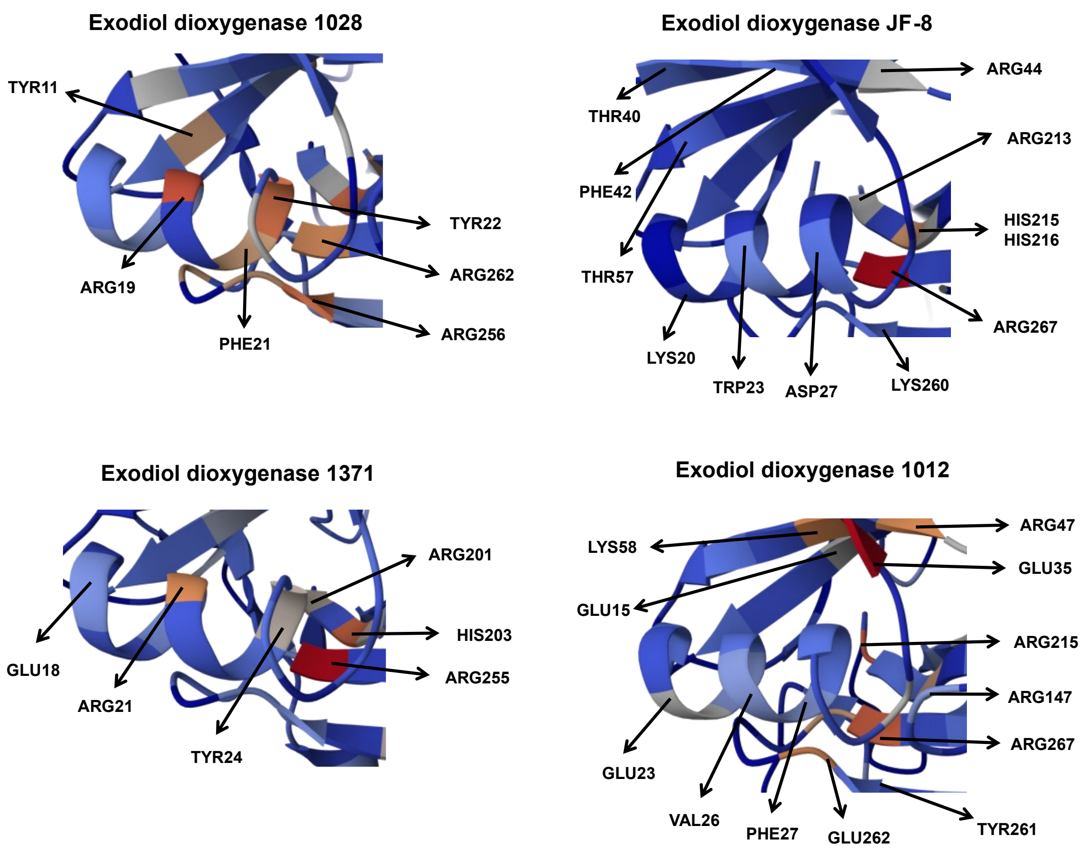

Fig. S8. Degree analysis of residues around α1 with stable backbone fluctuations in thermophilic EDOs 1012, 1028, 1371, and JF-8. The protein structures are presented in cartoon. The shift from dark blue to dark red indicates a gradual increase in residue degree.

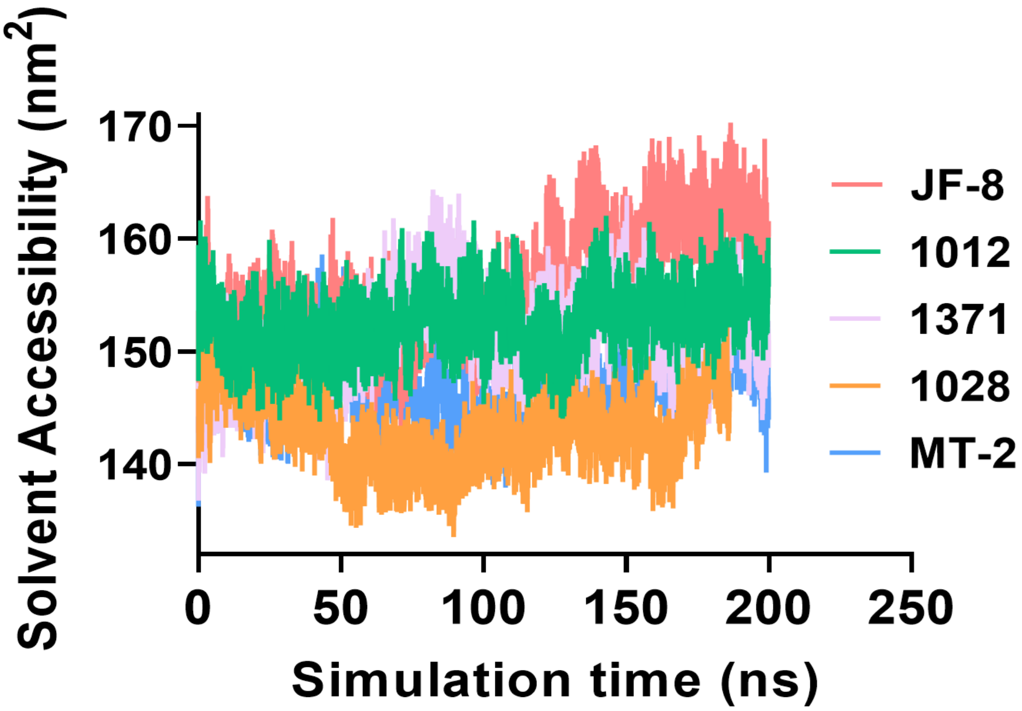

Fig. S9. Solvent accessibility analysis of mesophilic EDO MT-2, thermophilic EDOs JF-8, 1012, 1028, and 1371 via MD. Different EDOs are represented in curves of different colors.

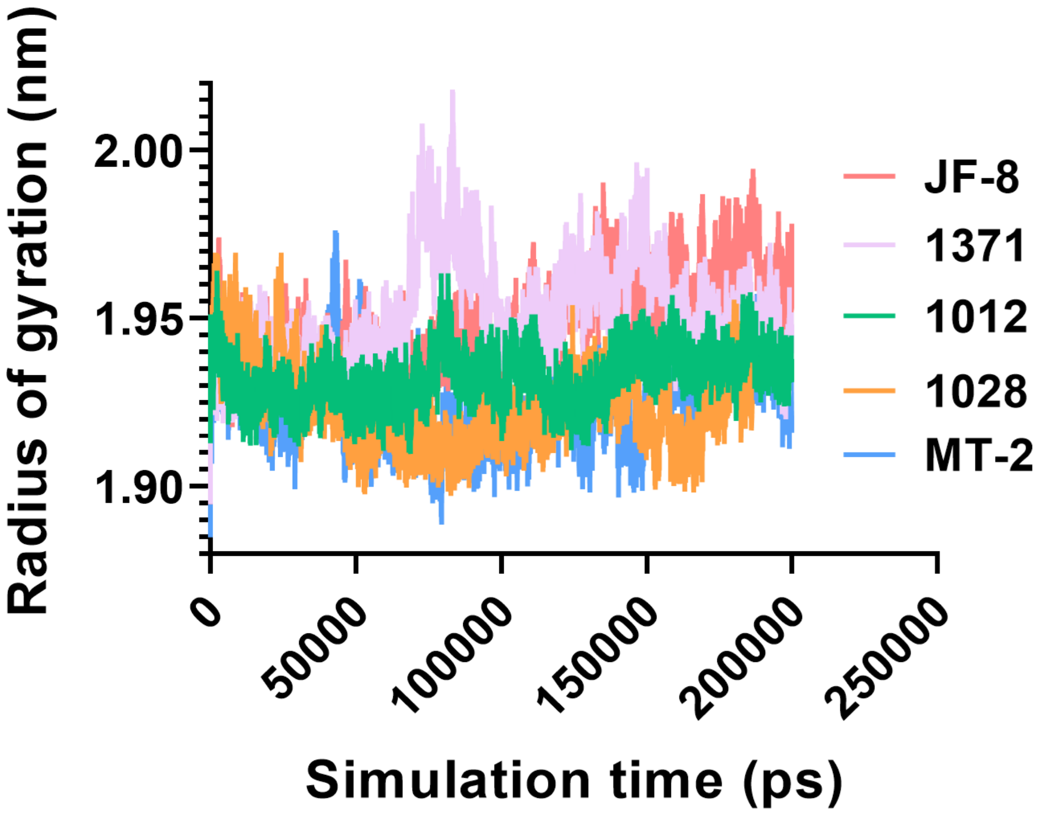

Fig. S10. Radius of gyration analysis of mesophilic EDO MT-2, thermophilic EDOs JF-8, 1012, 1028, and 1371 via MD. Different EDOs are represented in curves of different colors.

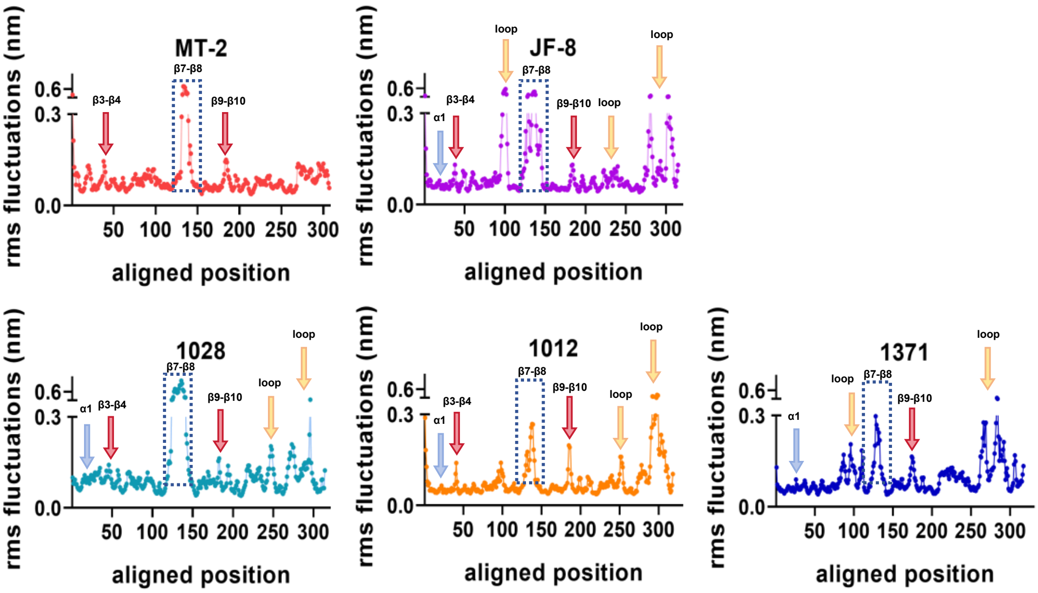

Fig. S11. Protein backbone fluctuations of mesophilic EDO MT-2, thermophilic EDOs JF-8, 1012, 1028, and 1371 via MD. The red arrows and blue dots box represent the common structural features of EDOs. The yellow arrows represent the more flexible structural features of thermophilic EDOs. The blue arrows represent the more stable structural features of thermophilic EDOs.

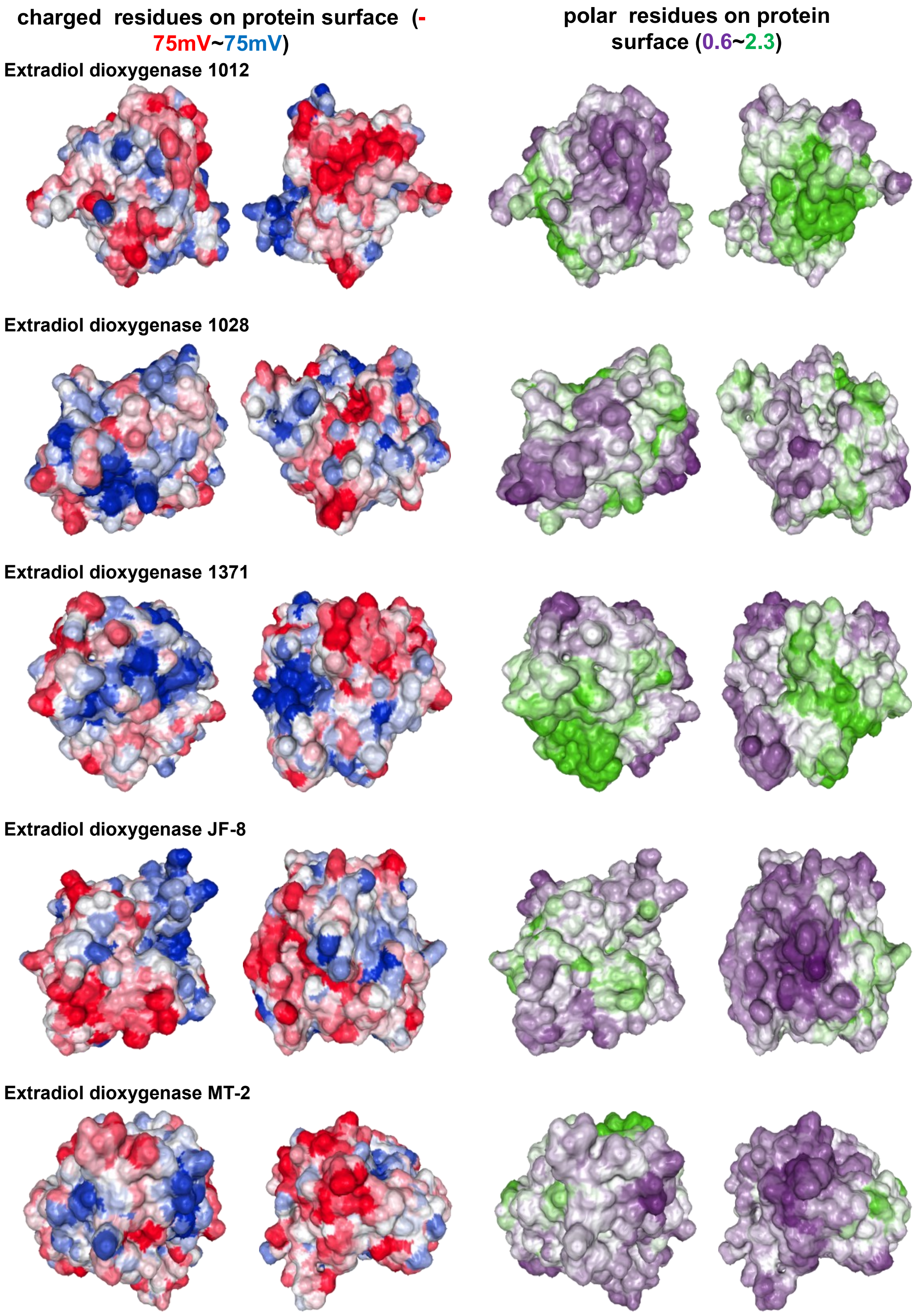

Fig. S12. Analysis of charged or polar features on protein surface of mesophilic EDO MT-2, thermophilic EDOs 1012,1371,1028, and JF-8. The two conformations of one protein are mirror images.

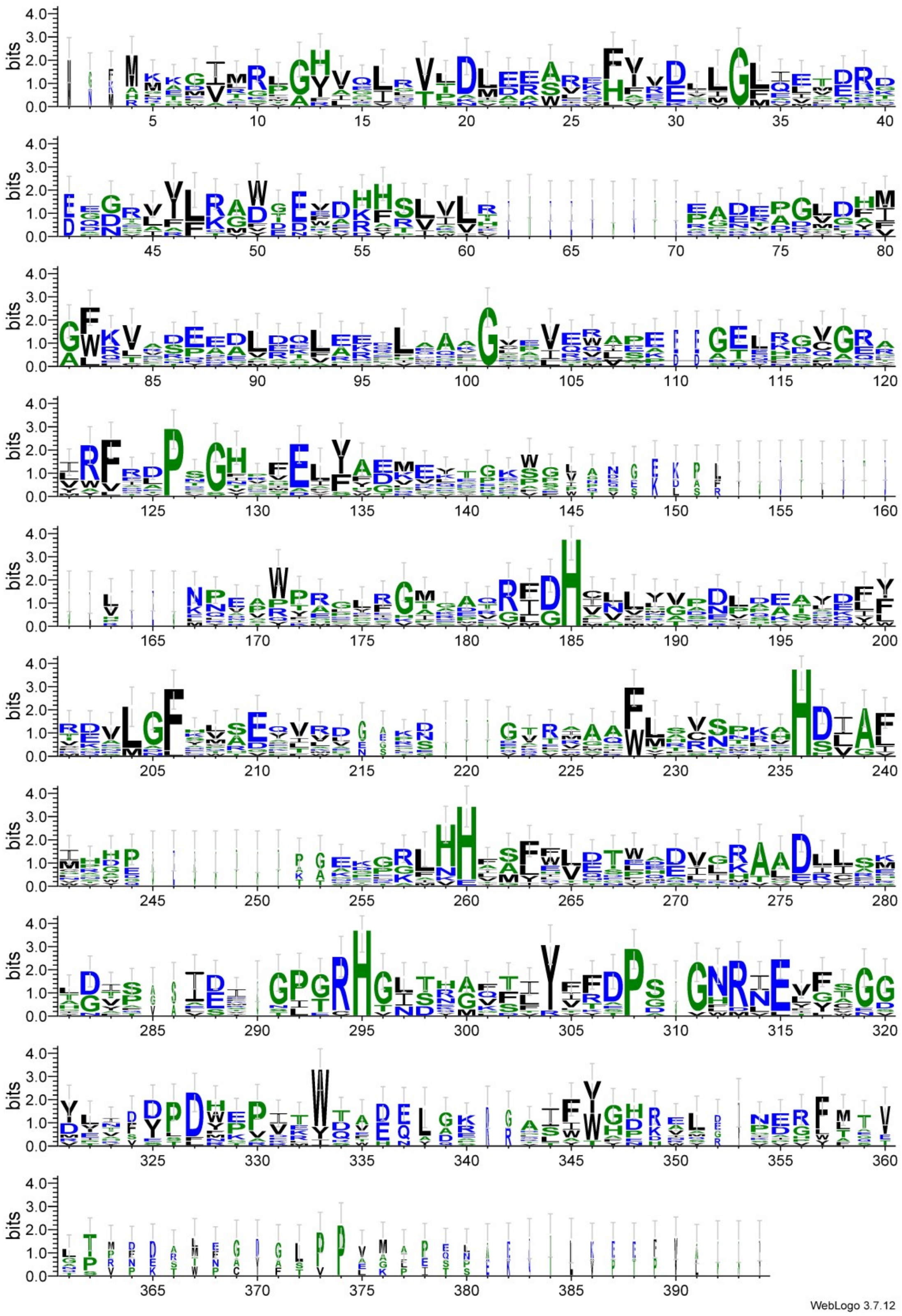

Fig. S13. Analysis of conserved sites of mesophilic EDO MT-2, thermophilic EDOs 1012,1371,1028, and JF-8.

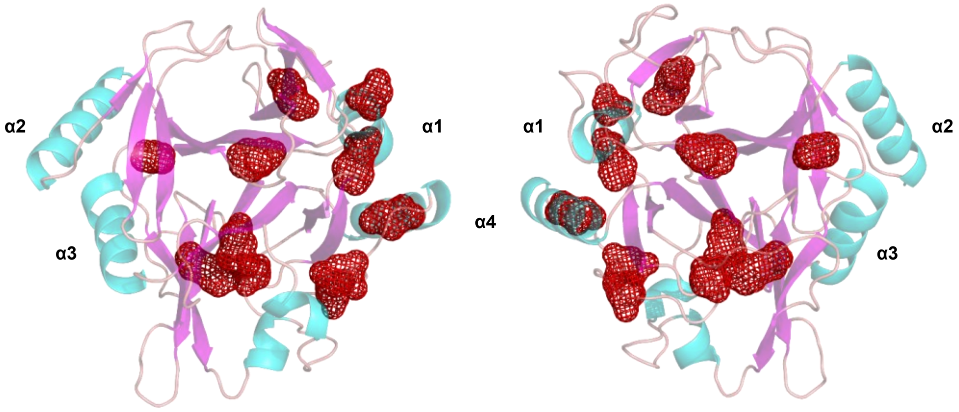

Fig. S14. Distribution of conserved site mutations in mesophilic EDOs MT-2. The protein structures are presented in cartoon. The two conformations of one protein are mirror images. The purple protein structure represents the β-sheet regions and loop, the blue protein structure represents the α-helix regions. Red clusters represent the mutation sites.

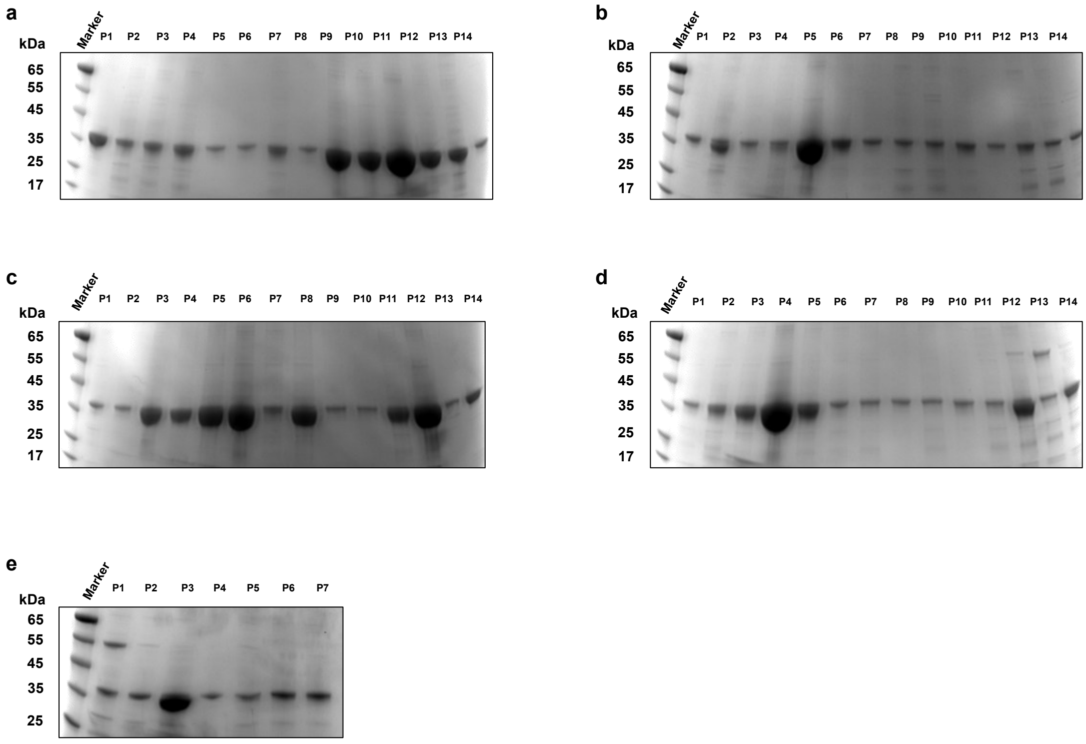

Fig. S15. Protein purification of mutations of mesophilic EDO MT-2. (a) P1-P14: MT2-1, MT2-3, MT2-2, MT2-4, G4Y, L13F, H24F, D51Y, A60F, G127Y, G145Y, A111F, and G114R. **(b)** P1-P14: S19E, K45R, F151I, T244G, G251A, I254L, Y274D, V5I, V280I, I232L, D240E, D271Y, H24F, T253F. **(c)** P1-P14: L172R, G173F, D230E, I254F, K20R, D84E, L85F, V92F, V171F, L176F, P243F, L306F, L78F, N273Y. **(d)** P1-P14: D66E, Q190F, E220Y, W222Y, A229F, L57F, V107F, V171F/L172R/G173F, D84E/V92F, N273Y/L306F, D51Y/D66E, L13F/L57F, A229F/I254F/P243F, L78F/L85F/L107F. **(e)** P1-P7: E220Y/W222Y/D230E, G127Y/G145Y, G4Y/A60F/H24F/K20R, L57F/V107F, V107F/A229F, A229F/L57F, L57F/V107F/A229F. The purified proteins are about 35 kDa.

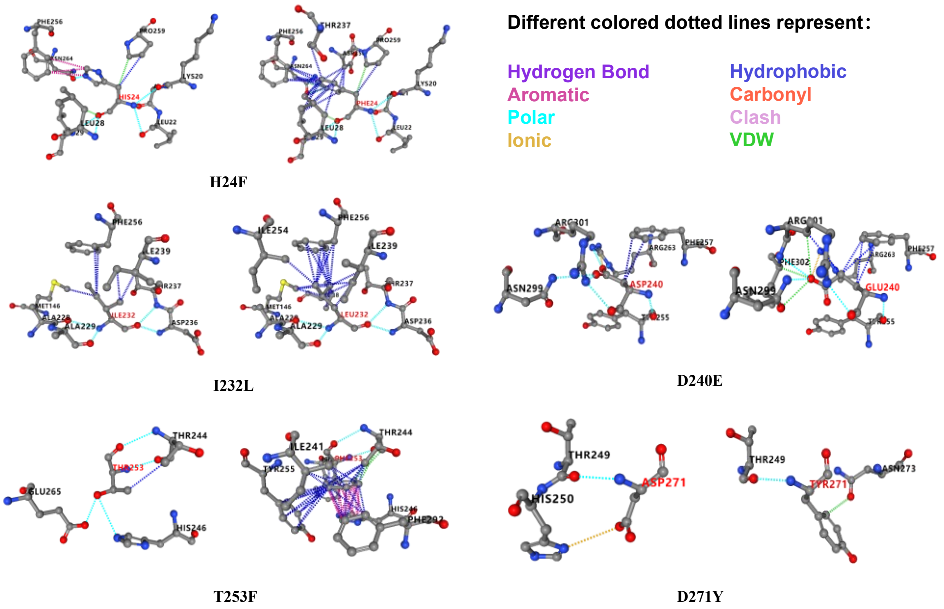

Fig. S16. The change of non-covalent bond connections of mutations H24F, I232L, D240E, T253F, and D271Y in mesophilic EDO MT-2. Different residues are in sticks conformation and the central residue interacts with different residues in the surrounding environment. Different colored dotted lines indicate non-covalent bonds.

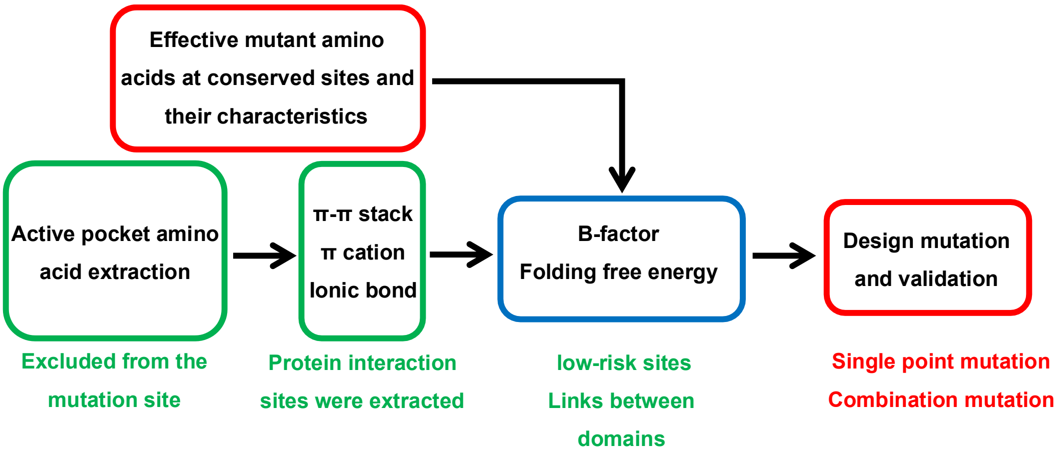

Fig. S17. Schematic diagram of the structure-guided mutation design.

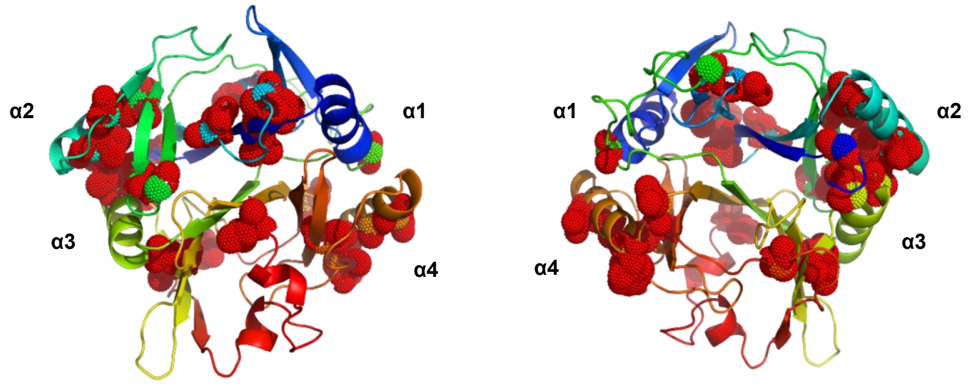

Fig. S18. Distribution of structure-guided mutations in mesophilic EDO MT-2. The protein structures are presented in cartoon. The two conformations of one protein are mirror images. The green line represents the protein structure. Red clusters represent the mutation sites.

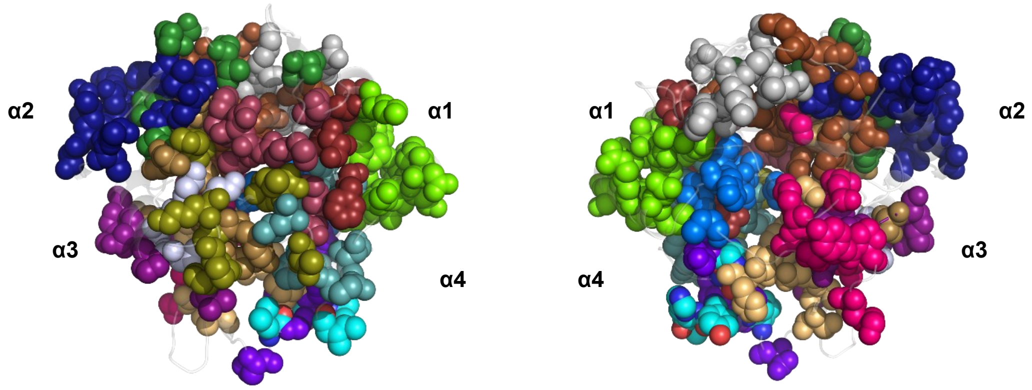

Fig. S19. Distribution of amino community in the non-covalent bond connection network of mesophilic EDO MT-2. The protein structure is shown in stick representation. The two conformations of one protein are mirror images. Communities are presented in different colors.

Exodiol dioxygenase MT-2

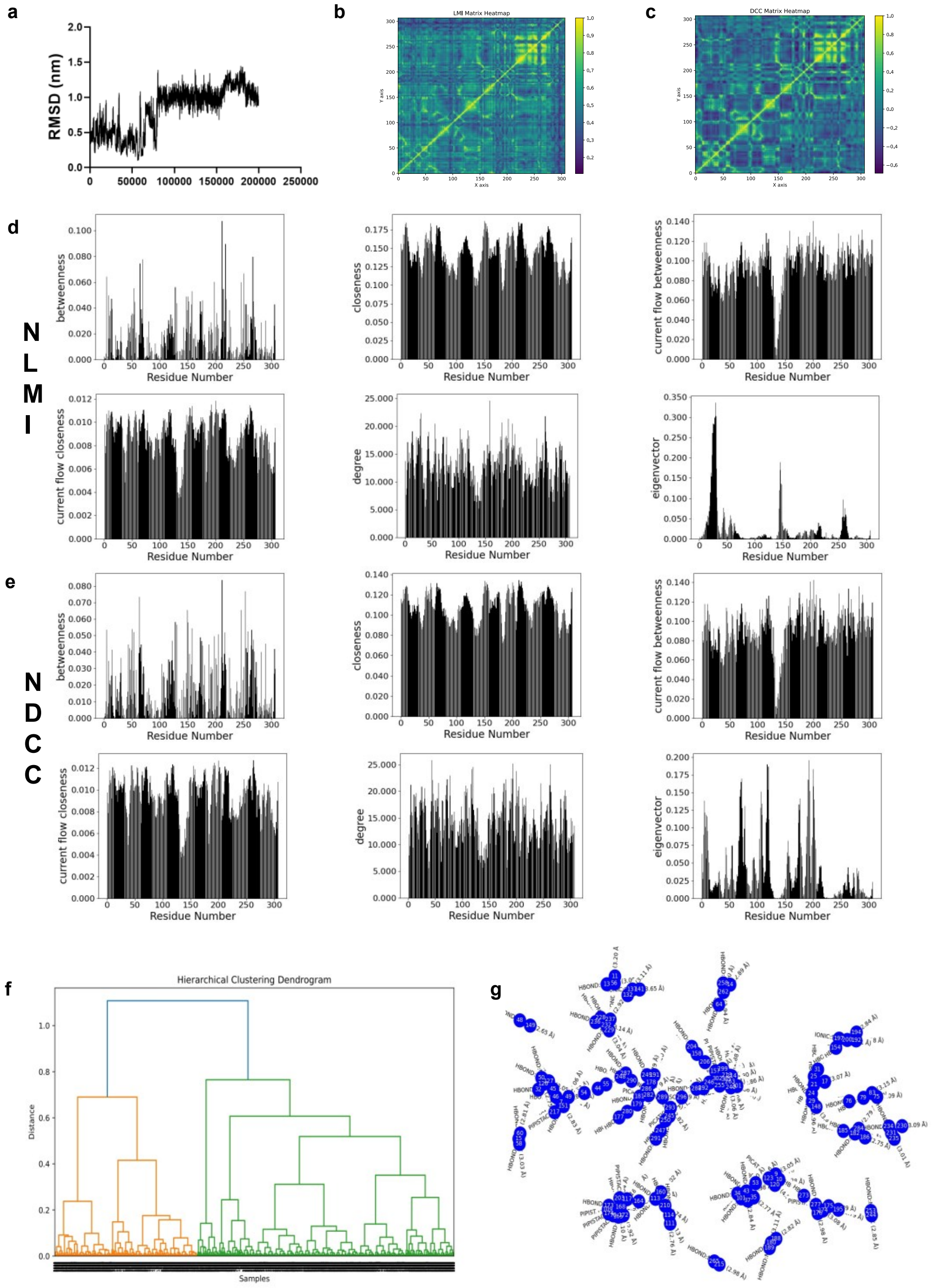

Fig. S20. Flow of cross-correlation network analysis for mesophilic EDO MT-2. **(a)** RMSD of protein via MD simulation. **(b)** Cross-correlation network of NLMI. **(c)** Cross-correlation network of NDCC. **(d)** Topological structure analysis of NLMI. **(e)** Network centrality analysis of NDCC. **(f)** Free energy landscape analysis. **(g)** Subnetwork of key structural amino acids.

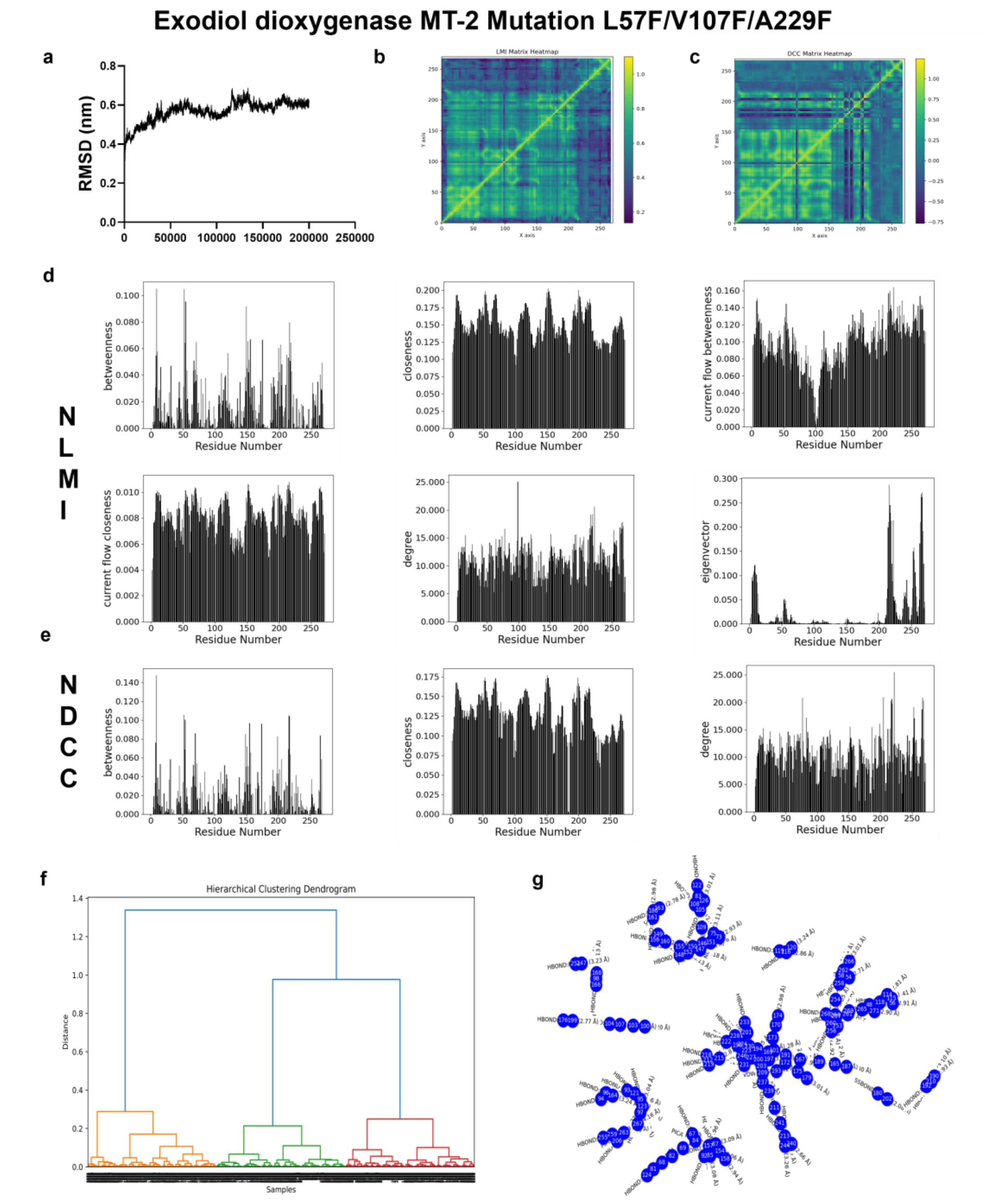

Fig. S21. Flow of cross-correlation network analysis for mutation A229F/L57F/V107F of mesophilic EDO MT-2. (a) RMSD of protein via MD simulation. **(b)** Cross-correlation network of NLMI. **(c)** Cross-correlation network of NDCC. **(d)** Topological structure analysis of NLMI. **(e)** Network centrality analysis of NDCC. **(f)** Free energy landscape analysis. **(g)** Subnetwork of key structural amino acids.

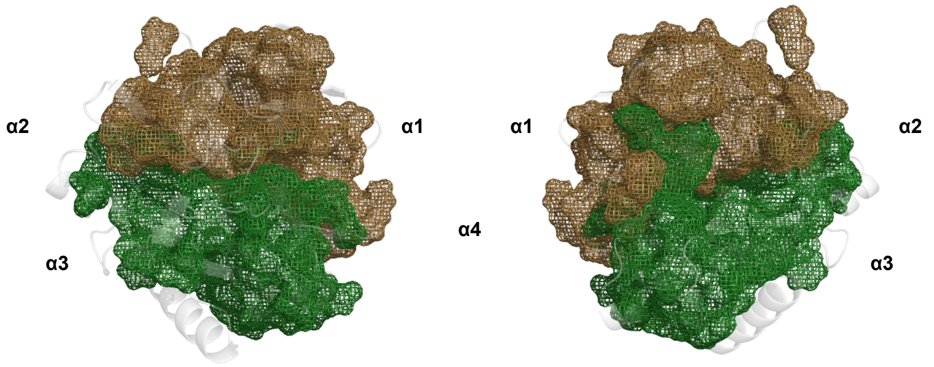

Fig. S22. The distribution of DSCLSs in thermophilic EDO 1012 from *Hydrogenibacillus* N12. The two conformations of one protein are mirror images. Different DSCLSs are shown in different colors.

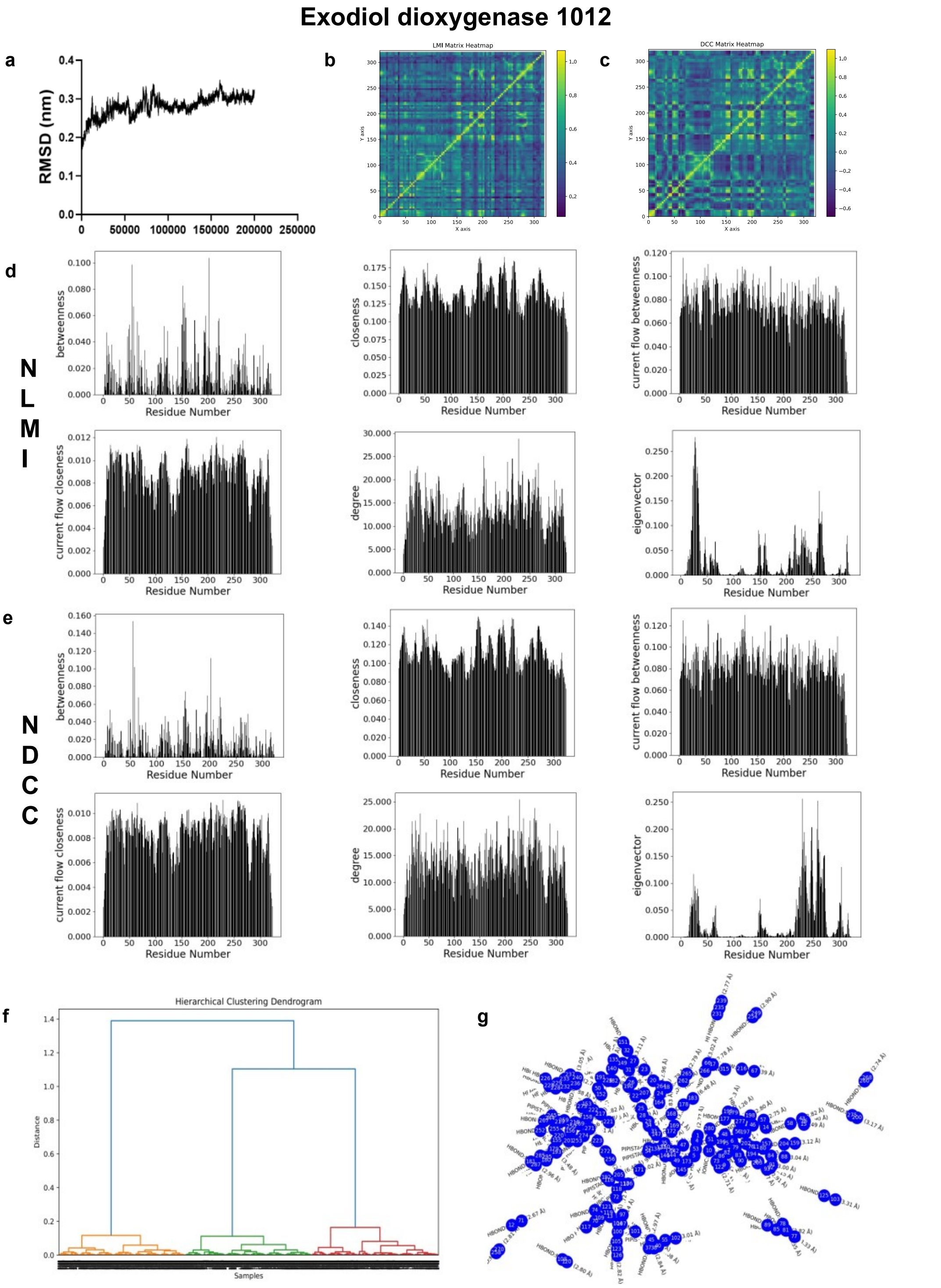

Fig. S23. Flow of cross-correlation network analysis for thermophilic EDO 1012. **(a)** RMSD of protein via MD simulation. **(b)** Cross-correlation network of NLMI. **(c)** Cross-correlation network of NDCC. **(d)** Topological structure analysis of NLMI. **(e)** Network centrality analysis of NDCC. **(f)** Free energy landscape analysis. **(g)** Subnetwork of key structural amino acids.

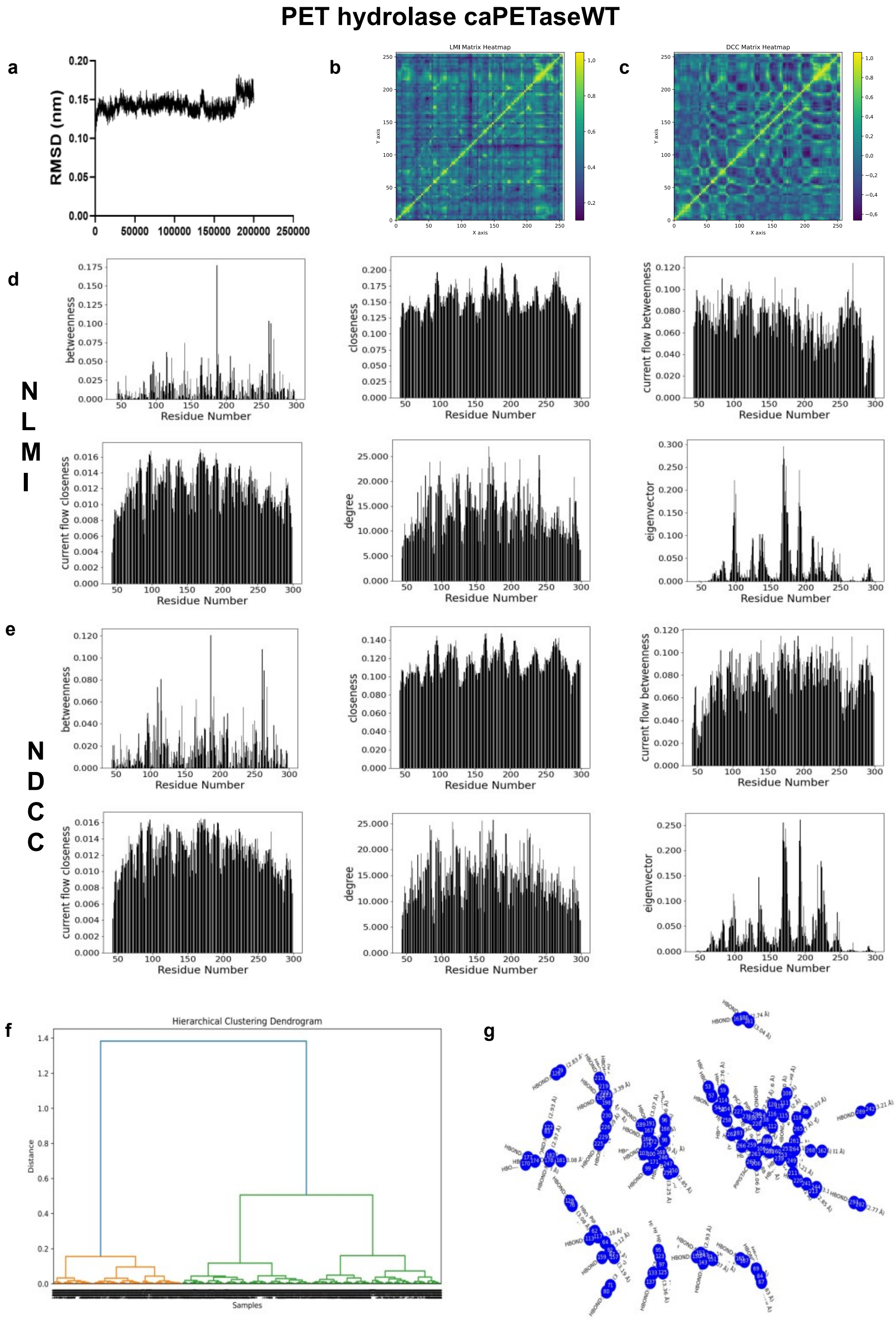

Fig. S24. Flow of cross-correlation network analysis for PET hydrolase caPETase^{WT}. (a) RMSD of protein via MD simulation. **(b)** Cross-correlation network of NLMI. **(c)** Cross-correlation network of NDCC. **(d)** Topological structure analysis of NLMI. **(e)** Network centrality analysis of NDCC. **(f)** Free energy landscape analysis. **(g)** Subnetwork of key structural amino acids.

PET hydrolase caPETaseM9

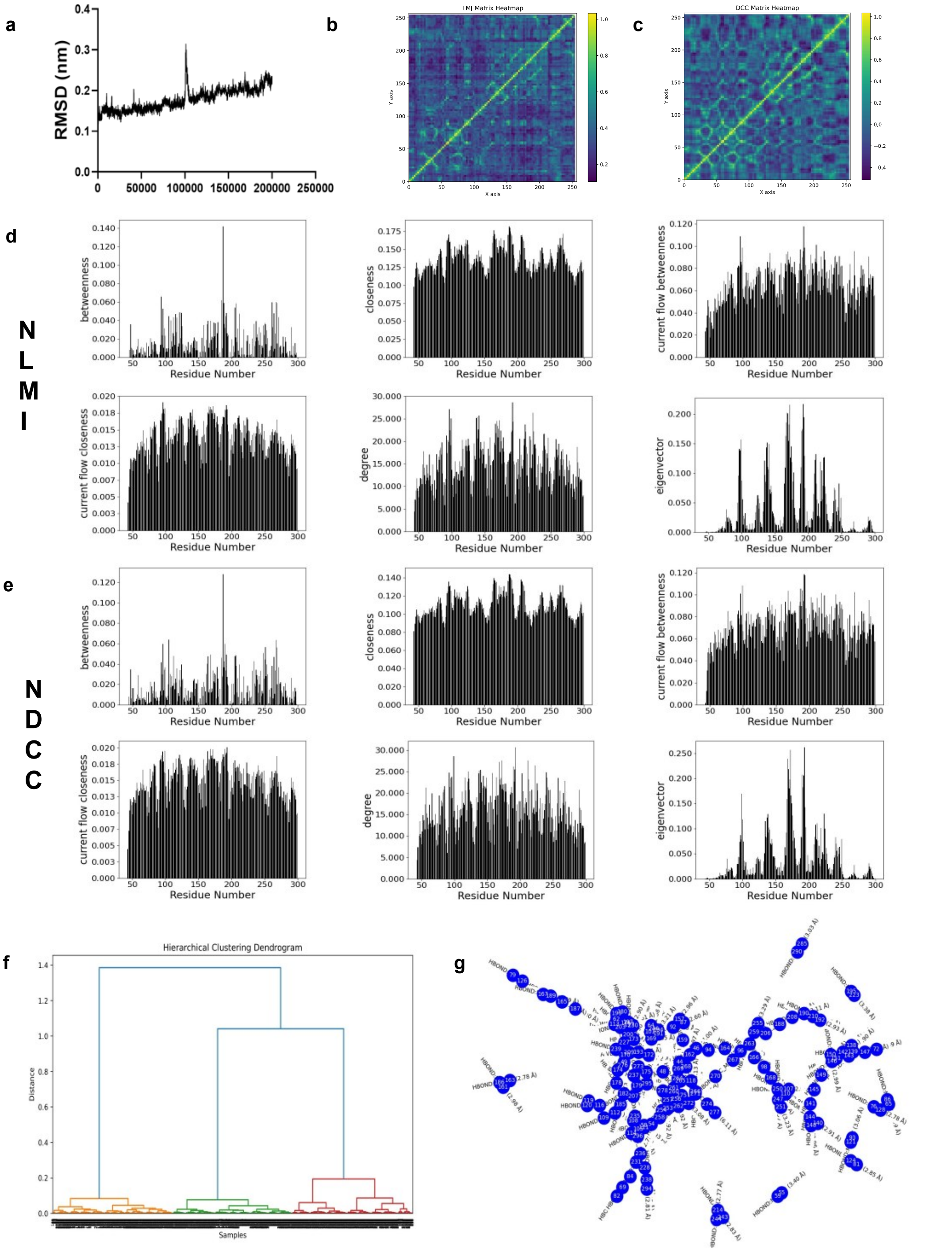

Fig. S25. Flow of cross-correlation network analysis for PET hydrolase mutant caPETase^{M9}. (a) RMSD of protein via MD simulation. **(b)** Cross-correlation network of NLMI. **(c)** Cross-correlation network of NDCC. **(d)** Topological structure analysis of NLMI. **(e)** Network centrality analysis of NDCC. **(f)** Free energy landscape analysis. **(g)** Subnetwork of key structural amino acids.

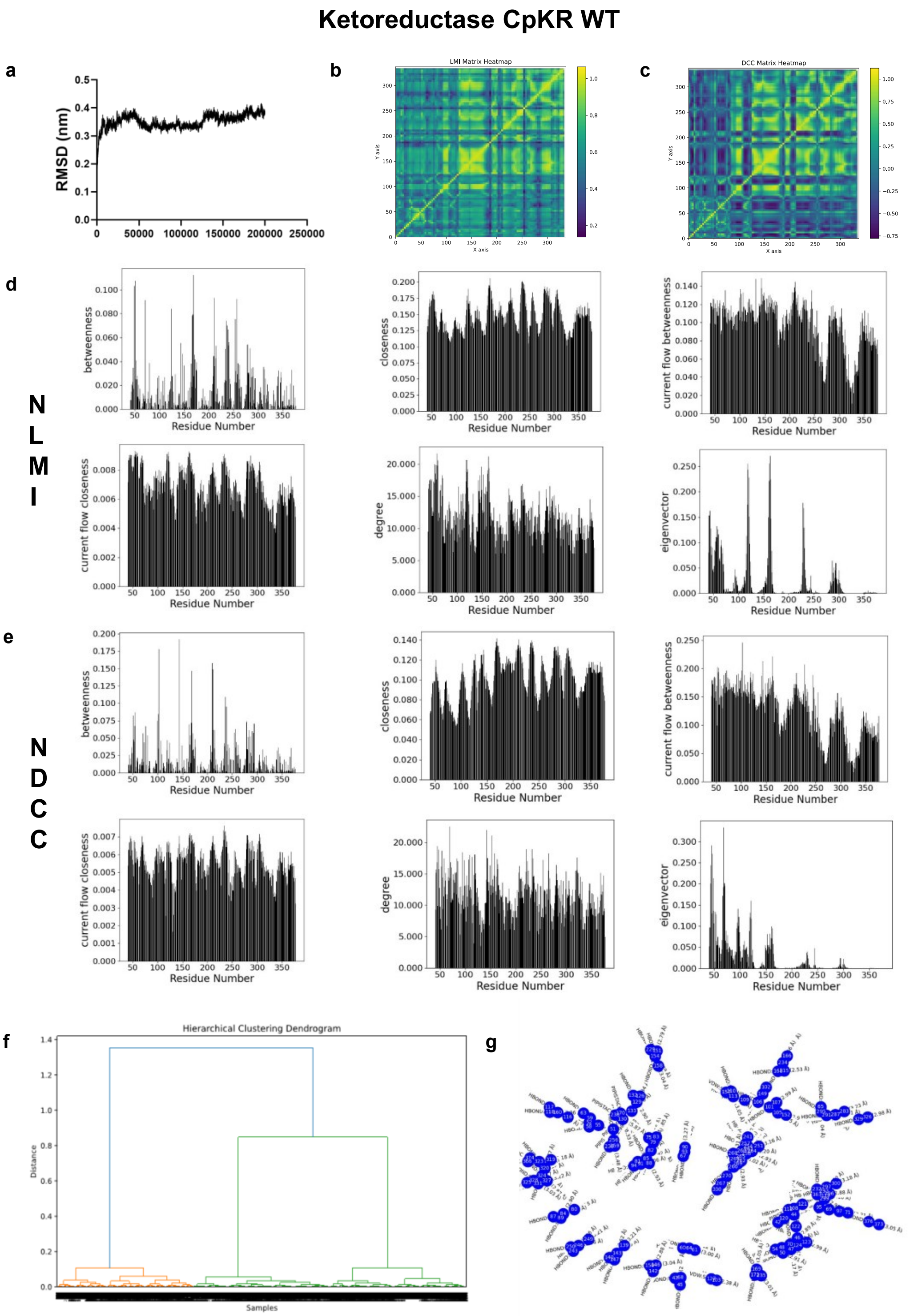

Fig. S26. Flow of cross-correlation network analysis for Ketoreductase CpKR ^{WT}. (a) RMSD of protein via MD simulation. **(b)** Cross-correlation network of NLMI. **(c)** Cross-correlation network of NDCC. **(d)** Topological structure analysis of NLMI. **(e)** Network centrality analysis of NDCC. **(f)** Free energy landscape analysis. **(g)** Subnetwork of key structural amino acids.

Ketoreductase CpKR M1

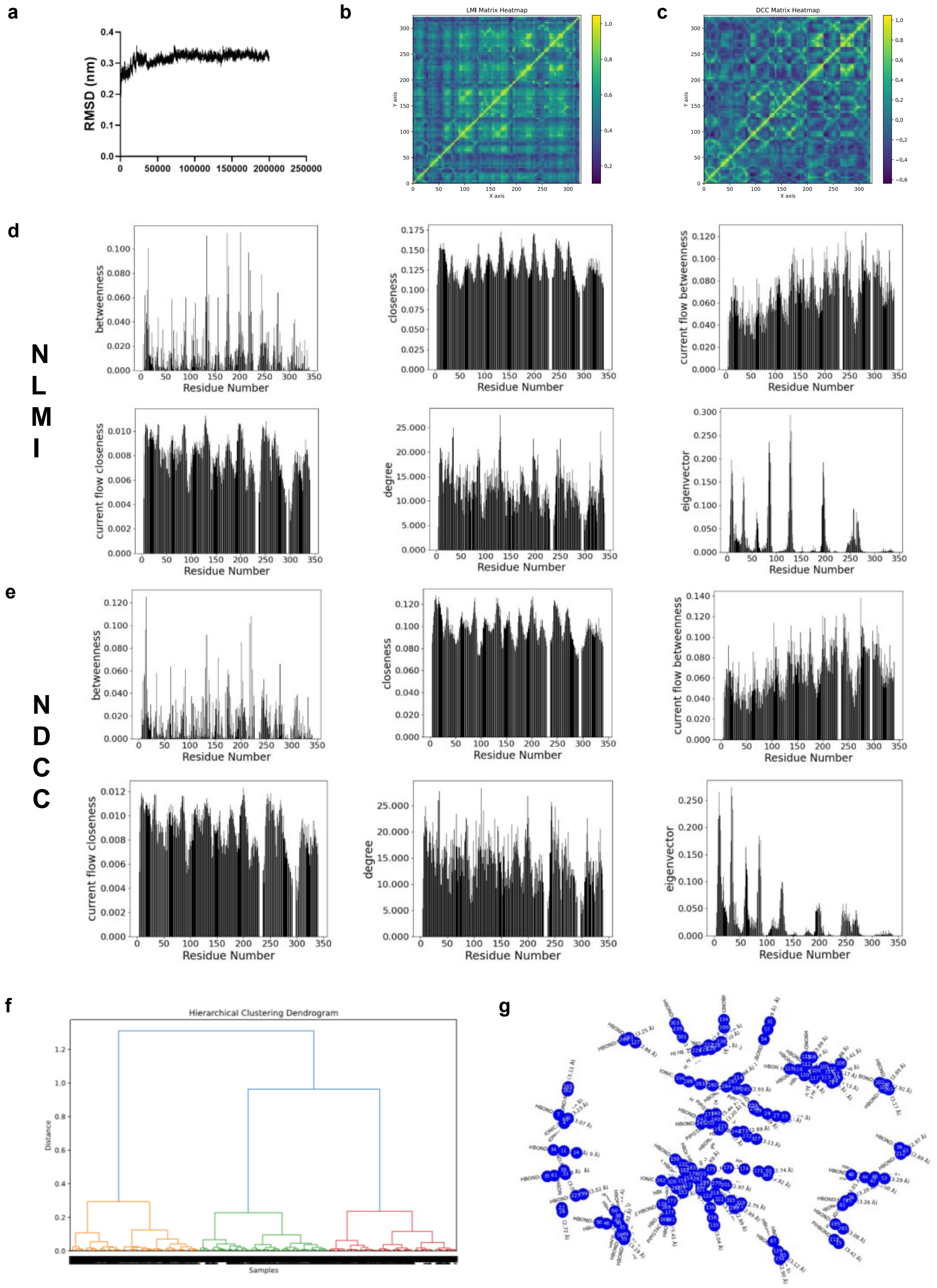

Fig. S27. Flow of cross-correlation network analysis for Ketoreductase CpKR ^{M1}. **(a)** RMSD of protein via MD simulation. **(b)** Cross-correlation network of NLMI. **(c)** Cross-correlation network of NDCC. **(d)** Topological structure analysis of NLMI. **(e)** Network centrality analysis of NDCC. **(f)** Free energy landscape analysis. **(g)** Subnetwork of key structural amino acids.

Exodiol dioxygenase L1

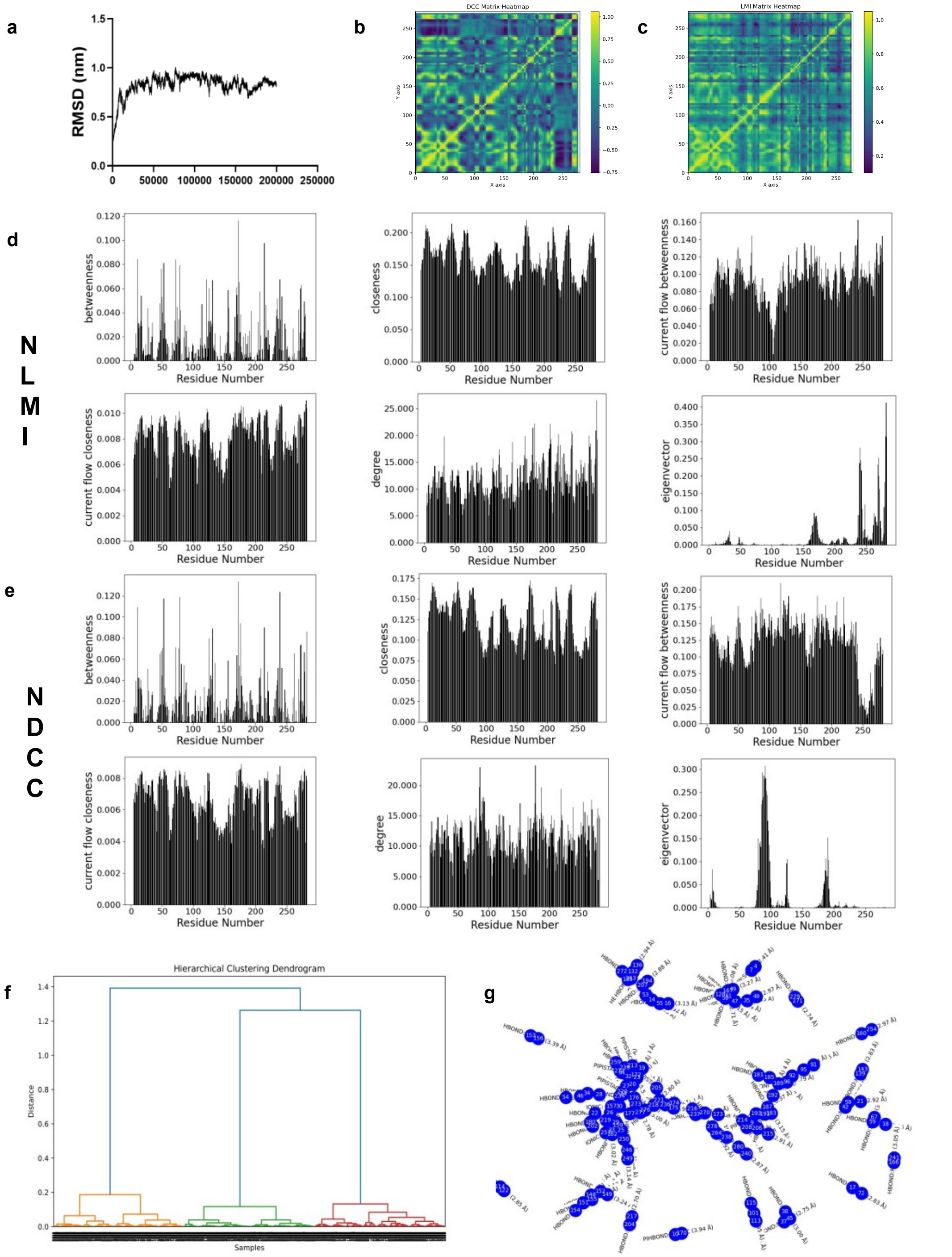

Fig. S28. Flow of cross-correlation network analysis for mesophilic EDO L1. (a) RMSD of protein via MD simulation. **(b)** Cross-correlation network of NLMI. **(c)** Cross-correlation network of NDCC. **(d)** Topological structure analysis of NLMI. **(e)** Network centrality analysis of NDCC. **(f)** Free energy landscape analysis. **(g)** Subnetwork of key structural amino acids.

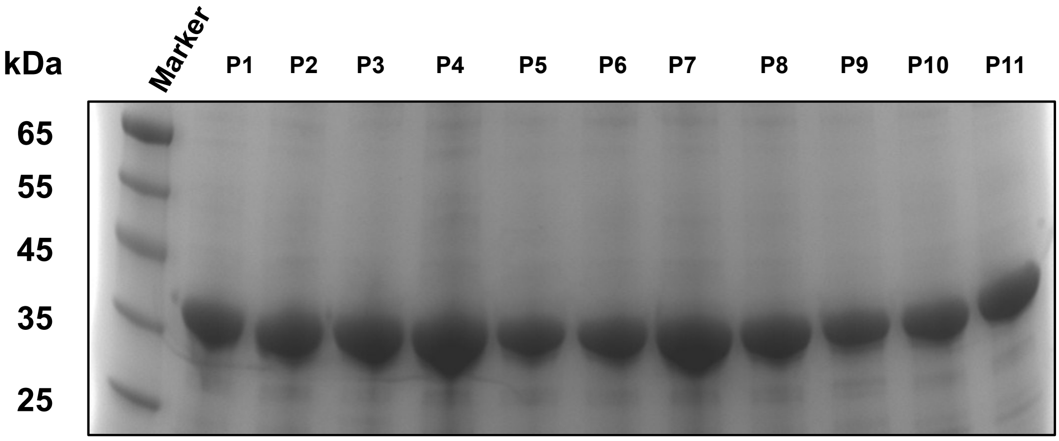

Fig. S29. Protein purification of mutations of mesophilic EDO L1. P1-P11: L1-WT, K236Y, I173F, L204F, V206F, P243R, I56F, A260R, S156E, T70Y, A227E. The purified proteins are about 35 kDa.

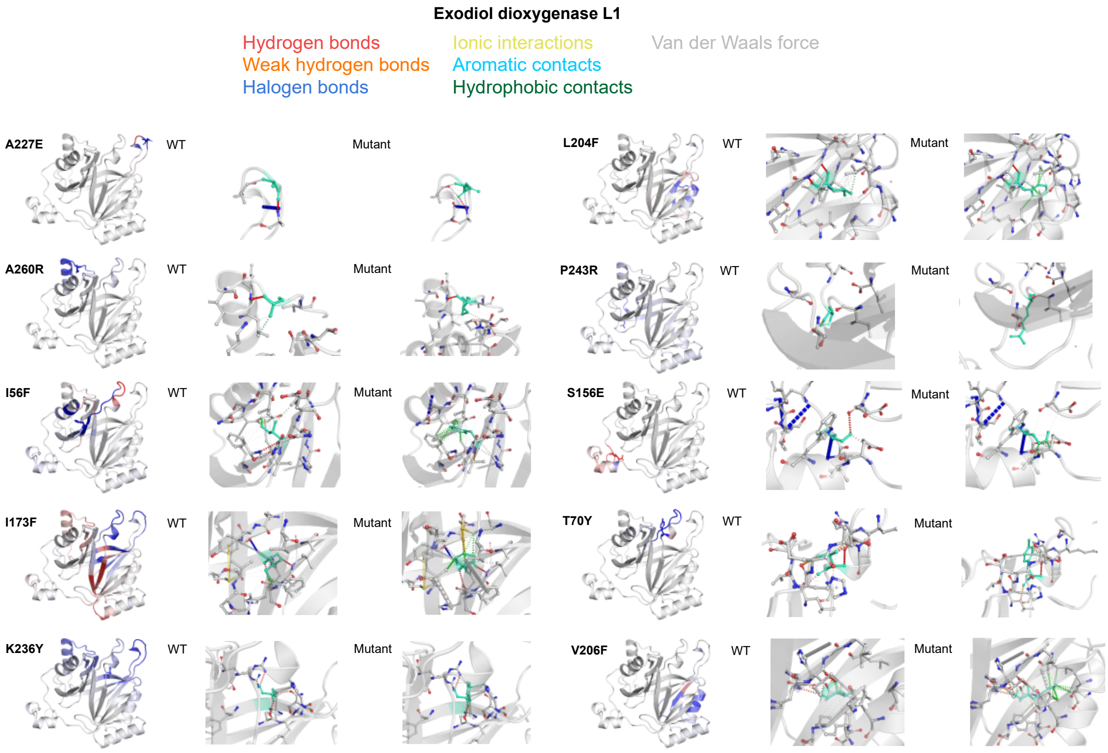

Fig. S30. Analysis of structural changes of mesophilic EDO L1 mutations. The protein structures are presented in cartoon. Different residues are in sticks conformation and the central residue interacts with different residues in the surrounding environment. Different colored dotted lines indicate non-covalent bonds. The protein's structural rigidity changes with mutations, with the blue region representing increased rigidity and the red region representing decreased rigidity.

Ectoine Synthetase xp-EctC

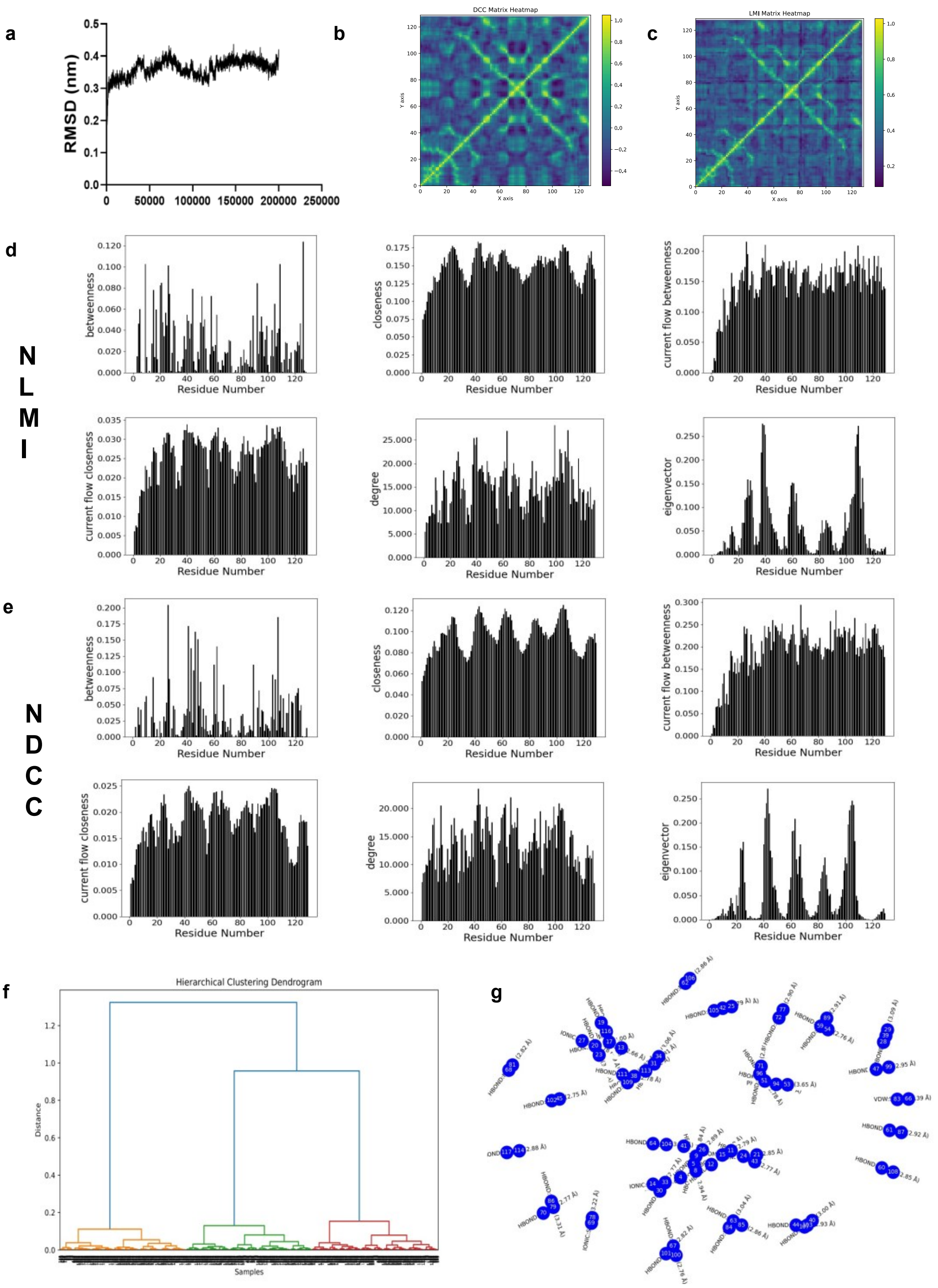

Fig. S31. Flow of cross-correlation network analysis for ectoine synthetase xp-EctC. (a) RMSD of protein via MD simulation. **(b)** Cross-correlation network of NLMI. **(c)** Cross-correlation network of NDCC. **(d)** Topological structure analysis of NLMI. **(e)** Network centrality analysis of NDCC. **(f)** Free energy landscape analysis. **(g)** Subnetwork of key structural amino acids.

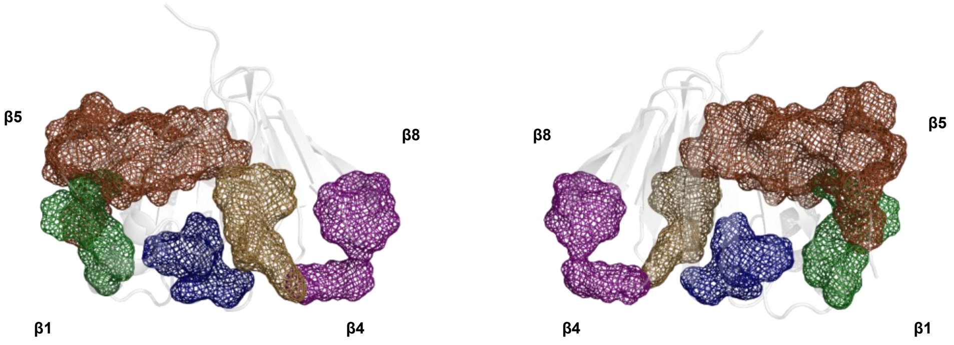

Fig. S32. The distribution of DSCLSs in thermophilic ectoine synthetase N12-EctC from *Hydrogenibacillus* N12. The two conformations of one protein are mirror images. Different DSCLSs are shown in different colors.

Ectoine Synthetase N12-EctC

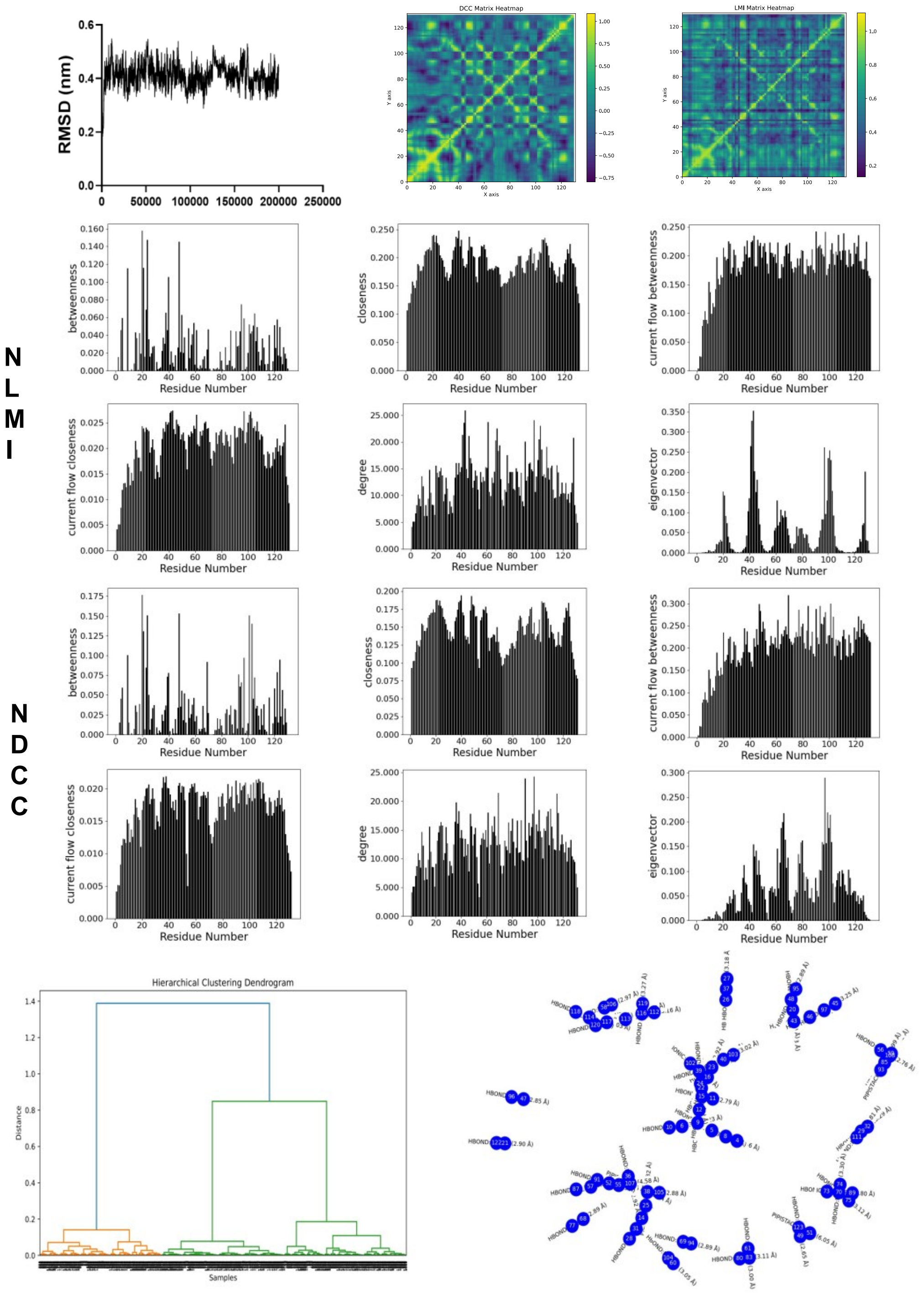

Fig. S33. Flow of cross-correlation network analysis for ectoine synthetase N12-EctC. (a) RMSD of protein via MD simulation. **(b)** Cross-correlation network of NLMI. **(c)** Cross-correlation network of NDCC. **(d)** Topological structure analysis of NLMI. **(e)** Network centrality analysis of NDCC. **(f)** Free energy landscape analysis. **(g)** Subnetwork of key structural amino acids.

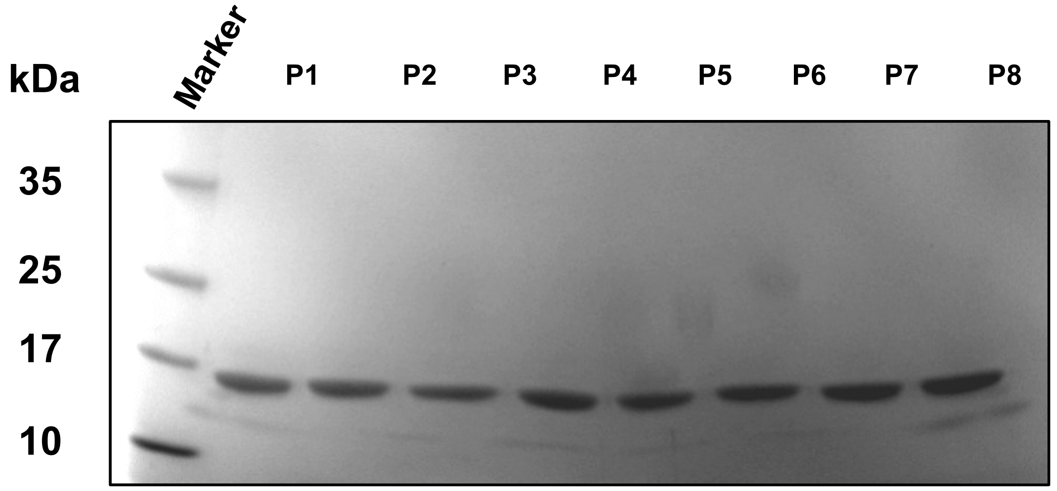

Fig. S34. Protein purification of mutations of ectoine synthetase xp-EctC. P1-P8: xp-EctC-WT, G91R, G123Y, V124R, R4D, L70F, N75R, I2R. The purified proteins are around 17 kDa.

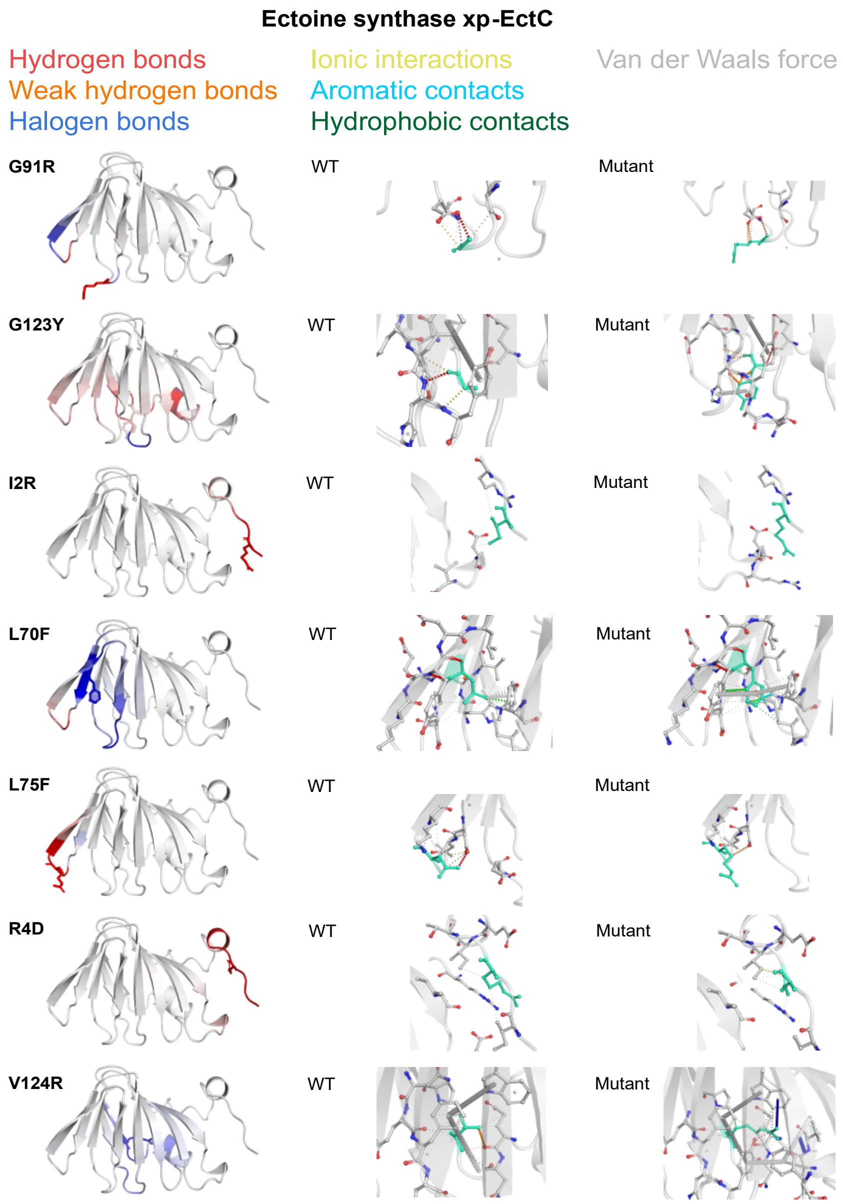

Fig. S35. Analysis of structural changes of ectoine synthetase xp-EctC mutations. The protein structures are presented in cartoon. Different residues are in sticks conformation and the central residue interacts with different residues in the surrounding environment. Different colored dotted lines indicate non-covalent bonds. The structural rigidity of the protein changes with mutations, with the blue structural part representing increased rigidity and the red structural part representing decreased rigidity.

Table S1. Percentage identification matrix of 13 selected EDOs

	**L1**	**R04**	**AAC**	**APD**	**A2**	**SEQ**	**YAA**	**MT-2**	**C23O1**	**JF-8**	**1012**	**1028**	**1371**
**L1**	100	15.25	17.24	16.74	18.33	15.29	19.01	18.6	17.77	17.55	21.37	22.73	18.72
**R04**	15.25	100	32.78	32.65	15.86	15.86	16.21	18.28	17.59	17.07	21.11	18.44	22.76
**AAC**	17.24	32.78	100	34.81	19.79	19.16	20.14	21.53	20.14	18.02	18.34	23.3	22.67
**APD**	16.74	32.65	34.81	100	16.07	18.28	18.57	17.5	16.79	18.28	17.61	18.32	22.7
**A2**	18.33	15.86	19.79	16.07	100	36.3	39.14	43.75	42.11	29.49	29.21	30.74	29.69
**SEQ**	15.29	15.86	19.16	18.28	36.3	100	47.21	47.87	49.18	28.81	26.71	26.78	27.49
**YAA**	19.01	16.21	20.14	18.57	39.14	47.21	100	72.96	78.5	27.95	27.3	28.62	25.94
**MT-2**	18.6	18.28	21.53	17.5	43.75	47.87	72.96	100	79.15	27.27	29.01	28.28	27.3
**C23O1**	17.77	17.59	20.14	16.79	42.11	49.18	78.5	79.15	100	26.94	27.65	26.6	26.28
**JF-8**	17.55	17.07	18.02	18.28	29.49	28.81	27.95	27.27	26.94	100	45.45	30.03	30.87
**1012**	21.37	21.11	18.34	17.61	29.21	26.71	27.3	29.01	27.65	45.45	100	32.44	35.08
**1028**	22.73	18.44	23.3	18.32	30.74	26.78	28.62	28.28	26.6	30.03	32.44	100	42.05
**1371**	18.72	22.76	22.67	22.7	29.69	27.49	25.94	27.3	26.28	30.87	35.08	42.05	100

Table S2. Detailed statistics of all amino acid types in 5 selected EDOs

Amino acid composition	1371		1028		1012		JF-8		MT-2	
Ala	28	8.60%	31	9.90%	26	8.00%	16	5.10%	21	6.80%
Arg	31	9.50%	35	11.10%	23	7.10%	20	6.30%	19	6.20%
Asn	5	1.50%	7	2.20%	7	2.20%	12	3.80%	9	2.90%
Asp	27	8.30%	20	6.40%	16	5.00%	20	6.30%	27	8.80%
Cys	3	0.90%	0	0.00%	0	0.00%	1	0.30%	3	1.00%
Gln	6	1.80%	6	1.90%	8	2.50%	9	2.90%	8	2.60%
Glu	24	7.40%	23	7.30%	31	9.60%	27	8.60%	21	6.80%
Gly	25	7.70%	24	7.60%	34	10.50%	30	9.50%	24	7.80%
His	13	4.00%	11	3.50%	11	3.40%	13	4.10%	15	4.90%
Ile	14	4.30%	13	4.10%	8	2.50%	22	7.00%	8	2.60%
Leu	33	10.10%	35	11.10%	33	10.20%	22	7.00%	34	11.10%
Lys	13	4.00%	3	1.00%	14	4.30%	17	5.40%	14	4.60%
Met	5	1.50%	5	1.60%	6	1.90%	8	2.50%	11	3.60%
Phe	17	5.20%	12	3.80%	17	5.30%	14	4.40%	16	5.20%
Pro	16	4.90%	22	7.00%	21	6.50%	21	6.70%	12	3.90%
Ser	11	3.40%	11	3.50%	14	4.30%	9	2.90%	10	3.30%
Thr	13	4.00%	13	4.10%	11	3.40%	15	4.80%	17	5.50%
Trp	7	2.10%	8	2.50%	11	3.40%	11	3.50%	5	1.60%
Tyr	15	4.60%	15	4.80%	11	3.40%	11	3.50%	12	3.90%
Val	20	6.10%	20	6.40%	21	6.50%	17	5.40%	21	6.80%
Pyl	0	0.00%	0	0.00%	0	0.00%	0	0.00%	0	0.00%
Sec	0	0.00%	0	0.00%	0	0.00%	0	0.00%	0	0.00%

Table S3. Detailed statistics of B-factor, free energy change, and alanine scanning data of structure-guide mutations in mesophilic EDO MT-2

	Position B-factor	Free energy change	Alanine scanning
**G4Y**	1.077	-3.4	0.12
**L13F**	0.233	-3.22	1.23
**H24F**	0.705	2.5	2.38
**D51Y**	0.81	6.13	0.67
**A60F**	0.878	-2.21	0.67
**G127Y**	2.003	-1.16	0.16
**G145Y**	1.019	-6.5	1.52
**A111F**	1.001	-6.55	0.43
**G114R**	1.932	-4.09	1.11
**L172R**	1.666	-5.57	1.66
**G173F**	1.205	-2.56	1.91
**D230E**	0.193	-2.22	1.12
**I254F**	0.209	-3.94	0.34
**K20R**	0.648	0.13	2.25
**D84E**	0.959	-1.76	1.16
**L85F**	1.098	-0.33	-0.02
**V92F**	0.928	-1.86	0.26
**V171F**	1.261	-2.91	0.34
**L176F**	0.741	0.82	0.44
**P243F**	0.323	1.63	1.73
**L306F**	1.473	-0.65	0.88
**L78F**	0.501	-0.27	1.3
**N273Y**	1.081	-5.48	0.02
**D66E**	0.469	1.06	0.61
**Q190F**	0.139	-0.56	1.37
**E220Y**	0.702	-6.12	0.5
**W222Y**	0.582	-1.51	-0.13
**A229F**	0.236	1.97	0.88
**L57F**	0.176	-5.79	1.83
**V107F**	0.191	-1.63	1.69

Table S4. Detailed statistics of free energy changes of mesophilic EDO L1 mutations via DynaMut

	WT	Mutant	Position	Prediction ΔΔG mCSM	Prediction ΔΔG SDM	Prediction ΔΔG DUET	Prediction ΔΔG ENCoM	ΔΔS ENCoM	ΔΔG DynaMut
1	S	E	156	-1.531 kcal/mol	0.19 kcal/mol	-1.217 kcal/mol	0.041 kcal/mol	-0.051 kcal.mol-1.K-1	**-0.59 kcal/mol**
2	P	R	243	0.032 kcal/mol	1.59 kcal/mol	0.397 kcal/mol	0.127 kcal/mol	-0.159 kcal.mol-1.K-1	**0.703 kcal/mol**
3	I	F	56	-1.871 kcal/mol	-1.54 kcal/mol	-2.113 kcal/mol	0.525 kcal/mol	-0.656 kcal.mol-1.K-1	**-0.956 kcal/mol**
4	V	F	206	-1.395 kcal/mol	-2.06 kcal/mol	-1.826 kcal/mol	0.457 kcal/mol	-0.572 kcal.mol-1.K-1	**-0.954 kcal/mol**
5	A	R	260	-0.715 kcal/mol	-2.33 kcal/mol	-0.87 kcal/mol	0.994 kcal/mol	-1.242 kcal.mol-1.K-1	**1.296 kcal/mol**
6	A	E	227	-0.78 kcal/mol	-0.16 kcal/mol	-0.421 kcal/mol	0.2 kcal/mol	-0.25 kcal.mol-1.K-1	**0.21 kcal/mol**
7	T	Y	70	-0.461 kcal/mol	-0.65 kcal/mol	-0.622 kcal/mol	0.526 kcal/mol	-0.657 kcal.mol-1.K-1	**1.022 kcal/mol**
8	I	F	173	-1.84 kcal/mol	-1.54 kcal/mol	-2.084 kcal/mol	0.36 kcal/mol	-0.45 kcal.mol-1.K-1	**-0.808 kcal/mol**
9	K	Y	236	0.183 kcal/mol	0.23 kcal/mol	0.293 kcal/mol	0.46 kcal/mol	-0.575 kcal.mol-1.K-1	**1.932 kcal/mol**
10	L	F	204	-1.411 kcal/mol	-0.75 kcal/mol	-1.416 kcal/mol	0.187 kcal/mol	-0.233 kcal.mol-1.K-1	**0.296 kcal/mol**

Table S5. Detailed statistics of free energy changes of ectoine synthetase xp-EctC mutations via DynaMut

	WT	Mutant	Position	Prediction ΔΔG mCSM	Prediction ΔΔG SDM	Prediction ΔΔG DUET	Prediction ΔΔG ENCoM	ΔΔS ENCoM	ΔΔG DynaMut
1	L	F	70	-1.545 kcal/mol	-0.66 kcal/mol	-1.513 kcal/mol	0.168 kcal/mol	-0.21 kcal.mol-1.K-1	**0.237 kcal/mol**
2	G	Y	123	-0.59 kcal/mol	-3.29 kcal/mol	-1.443 kcal/mol	-0.22 kcal/mol	0.274 kcal.mol-1.K-1	**-1.16 kcal/mol**
3	N	R	75	0.138 kcal/mol	0.1 kcal/mol	0.363 kcal/mol	-0.043 kcal/mol	0.054 kcal.mol-1.K-1	**0.268 kcal/mol**
4	V	R	124	-1.263 kcal/mol	-3.69 kcal/mol	-1.382 kcal/mol	0.643 kcal/mol	-0.804 kcal.mol-1.K-1	**0.1 kcal/mol**
5	R	D	4	0.067 kcal/mol	-0.31 kcal/mol	0.16 kcal/mol	-0.241 kcal/mol	0.301 kcal.mol-1.K-1	**-0.171 kcal/mol**
6	I	R	2	0.107 kcal/mol	-1.29 kcal/mol	0.158 kcal/mol	0.065 kcal/mol	-0.082 kcal.mol-1.K-1	**0.001 kcal/mol**
7	G	R	91	-0.376 kcal/mol	0.18 kcal/mol	-0.255 kcal/mol	-0.029 kcal/mol	0.037 kcal.mol-1.K-1	**-0.126 kcal/mol**

Table S6. Protein mutation primers

primer	sequence(5'to3')
1012-F	GACCATGGAAATGGGCAAACGCCGGCACTG
1012-R	GACTCGAGGCTCTGCGGCAGCATGACCG
1028-F	GACCATGGAAATGATCCTCCGCTTGGGC
1028-R	GACTCGAGCCCCGGCCAGCGGTC
1371-F	GACCATGGAAATGGGCGACATCATCCGC
1371-R	GACTCGAGGAGGACGAAGGTCGGCT
MT2-4-1	GACCCGGACGCATAACATATTTATTCATTTCCATGG
MT2-4-2	CCATGGAAATGAATAAATATGTTATGCGTCCGGGTCA
MT2-60-1	CATAAAATCCATACCCGGTTCATCGAATTCACGCAGAACCAGGCTAAATTT
MT2-60-2	AAATTTAGCCTGGTTCTGCGTGAATTCGATGAACCGGGTATGGATTTTATG
MT2-24-1	CCAGCAGCTCAACATAATATTCCAGTGCTTTGCTCAT
MT2-24-2	ATGAGCAAAGCACTGGAATATTATGTTGAGCTGCTGG
MT2-20-1	ATGTTCCAGTGCTCTGCTCATATCCAGAACACGCA
MT2-20-2	TGCGTGTTCTGGATATGAGCAGAGCACTGGAACAT
MT2-13-1	CTCATATCCAGAACACGGAACTGAACATGACCCGGAC
MT2-13-2	GTCCGGGTCATGTTCAGTTCCGTGTTCTGGATATGAG
MT2-57-1	CCGGTTCATCTGCTTCACGGAAAACCAGGCTAAATTTATCAACT
MT2-57-2	AGTTGATAAATTTAGCCTGGTTTTCCGTGAAGCAGATGAACCGG
MT2-51-1	CAGAACCAGGCTAAATTTATAAACTTCGGTCCATGCTTTCA
MT2-51-2	TGAAAGCATGGACCGAAGTTTATAAATTTAGCCTGGTTCTG
MT2-66-1	CCACTTTAAAACCCATAAACTCCATACCCGGTTCATCTG
MT2-66-2	CAGATGAACCGGGTATGGAGTTTATGGGTTTTAAAGTGG
MT2-84-1	CAACCATATGCCATCAGCTCACGTTCCAGCTGACG
MT2-84-2	CGTCAGCTGGAACGTGAGCTGATGGCATATGGTTG
MT2-92-1	GCCGGCAGCTGTTCAAATGCACAACCATATGCC
MT2-92-2	GGCATATGGTTGTGCATTTGAACAGCTGCCGGC
MT2-78-1	GTTCCAGCTGACGGAATGCGTCCTCGTCCAC
MT2-78-2	GTGGACGAGGACGCATTCCGTCAGCTGGAAC
MT2-85-1	CAACCATATGCCATGAAATCACGTTCCAGCTGACGCAGT
MT2-85-2	ACTGCGTCAGCTGGAACGTGATTTCATGGCATATGGTTG
MT2-107-1	GGTGCCTGAAAACGAAAACGACGACCACAGCTA
MT2-107-2	TAGCTGTGGTCGTCGTTTTCGTTTTCAGGCACC
MT2-111-1	CAAAATGATGACCGCTCGGGAACTGAAAACGAACACGACGACCAC

MT2-111-2	GTGGTCGTCGTGTTCGTTTTCAGTTCCCGAGCGGTCATCATTTTG
MT2-114-1	CAGTTCAAAATGATGACGGCTCGGTGCCTGAAAA
MT2-114-2	TTTTCAGGCACCGAGCCGTCATCATTTTGAACTG
MT2-127-1	GATTAACATCATTCAGACCCCACTTATAGGTGTACTCTTTATCTGCATACAGT
MT2-127-2	ACTGTATGCAGATAAAGAGTACACCTATAAGTGGGGTCTGAATGATGTTAATC
MT2-145-1	GAACTGCTGCCATATATTTCAGATCACGCGGCCATGCTTC
MT2-145-2	GAAGCATGGCCGCGTGATCTGAAATATATGGCAGCAGTTC
MT2-171-1	GCCAGGTAAAAACCCAGAAATTTGGTAAACAGATCATAGGT
MT2-171-2	ACCTATGATCTGTTTACCAAATTTCTGGGTTTTTACCTGGC
MT2-172-1	TCTGCCAGGTAAAAACCCCGAACTTTGGTAAACAGATC
MT2-172-2	GATCTGTTTACCAAAGTTCGGGGTTTTTACCTGGCAGA
MT2-173-1	GAACCTGTTCTGCCAGGTAAAAAAACAGAACTTTGGTAAACAGATCAT
MT2-173-2	ATGATCTGTTTACCAAAGTTCTGTTTTTTTACCTGGCAGAACAGGTTC
MT2-176-1	CATCCAGAACCTGTTCTGCGAAGTAAAAACCCAGAACTTTG
MT2-176-2	CAAAGTTCTGGGTTTTTACTTCGCAGAACAGGTTCTGGATG
MT2-190-1	TTTGGTGCTCAGGCTCAGAAAGAATGCAACACGGGTACCATTTTC
MT2-190-2	GAAAATGGTACCCGTGTTGCATTCTTTCTGAGCCTGAGCACCAAA
MT2-220-1	GCAGCAGATCTTCCCAGGTATACAGATGAAAGCTAACATGA
MT2-220-2	TCATGTTAGCTTTCATCTGTATACCTGGGAAGATCTGCTGC
MT2-222-1	CTGCACGCAGCAGATCTTCATAGGTTTCCAGATGAAAGCT
MT2-222-2	AGCTTTCATCTGGAAACCTATGAAGATCTGCTGCGTGCAG
MT2-230-1	GGTCATGCTAATCAGCTCTGCTGCACGCAGC
MT2-230-2	GCTGCGTGCAGCAGAGCTGATTAGCATGACC
MT2-229-1	TCGGTCATGCTAATCAGATCGAATGCACGCAGCAGATCTTCCC
MT2-229-2	GGGAAGATCTGCTGCGTGCATTCGATCTGATTAGCATGACCGA
MT2-254-1	TCGGGTCGAAAAAATAAAAGGTTTTACCGTGGGTCAG
MT2-254-2	CTGACCCACGGTAAAACCTTTTATTTTTTCGACCCGA
MT2-243-1	GGGTCAGACCATGACGGGTGAAGCCAATATCAATGCTGGTA
MT2-243-2	TACCAGCATTGATATTGGCTTCACCCGTCATGGTCTGACCC
MT2-273-1	GGTTTATGGTCCGGGTAATAGTAATCACCACCACAAAAAACTTCATTA
MT2-273-2	TAATGAAGTTTTTTGTGGTGGTGATTACTATTACCCGGACCATAAACC
MT2-306-1	GTGCTCGAGGGTGAAAACGGTCATAAAGCGCTCG
MT2-306-2	CGAGCGCTTTATGACCGTTTTCACCCTCGAGCAC
xp-N75R-F	CGAGATCGTATTCCTTGTTCCTGTCCAGGTCGATCAGCTT
xp-N75R-R	AAGCTGATCGACCTGGACAGGAACAAGGAATACGATCTCG

xp-G91R-F	GTGACGCTCGTGACGGTTGAGCAGGTACA
xp-G91R-R	TGTACCTGCTCAACCGTCACGAGCGTCAC
xp-I2R-F	CTCGGTGGTGGTGCGAACCCTCATGGTATATCTCCTTC
xp-I2R-R	GAAGGAGATATACCATGAGGGTTCGCACCACCACCGAG
xp-R4D-F	CTCGGTGGTGGTGTCAACGATCATGGTATATCTCCTTCT
xp-R4D-R	AGAAGGAGATATACCATGATCGTTGACACCACCACCGAG
xp-G123Y-F	CCACCAGTGGGTAGACATAGTTCTCGTCGTGTACCT
xp-G123Y-R	AGGTACACGACGAGAACTATGTCTACCCACTGGTGG
xp-L70F-F	GTTGTCCAGGTCGATGAACTTGCCGGTGCCCTC
xp-L70F-R	GAGGGCACCGGCAAGTTCATCGACCTGGACAAC
xp-V124R-F	TCCACCAGTGGGTAGCGACCGTTCTCGTCGTG
xp-V124R-R	CACGACGAGAACGGTCGCTACCCACTGGTGGA
L1-I56F-F	CATTCTCTTCGTGTTCAATAAAAACTAACGGGGTATTACCATC
L1-I56F-R	GATGGTAATACCCCGTTAGTTTTTATTGAACACGAAGAGAATG
L1-A260R-F	CTTCGCTAACCGGGCGATTAATTTTTTTCAGCTGTTCCAGAAC
L1-A260R-R	GTTCTGGAACAGCTGAAAAAAATTAATCGCCCGGTTAGCGAAG
L1-I173F-F	ACATGCAGATGAAAATGACCCATAACGGTATCGCTC
L1-I173F-R	GAGCGATACCGTTATGGGTCATTTTCATCTGCATGT
L1-V206F-F	GTGATATTTATTGCTTGCAAAAACAGTGCCTGATGACGAAAC
L1-V206F-R	GTTTCGTCATCAGGCACTGTTTTTGCAAGCAATAAATATCAC
L1-L204F-F	GATATTTATTGCTTGCAACAAAGAATGCCTGATGACGAAACGGAACG
L1-L204F-R	CGTTCCGTTTCGTCATCAGGCATTCTTTGTTGCAAGCAATAAATATC
L1-L21F-F	AAACTGCTGGCTACGTTCGAAATCGCTAACCAGCAGATG
L1-L21F-R	CATCTGCTGGTTAGCGATTTCGAACGTAGCCAGCAGTTT
L1-T70Y-F	GCAAAATGATATAAACCTGTATAACGCGGACGTTTCGGCTGGGC
L1-T70Y-R	GCCCAGCCGAAACGTCCGCGTTATACAGGTTTATATCATTTTGC
L1-A227E-F	CTATTTGCTGCCGGTTCAGGTGCACCTTCAC
L1-A227E-R	GTGAAGGTGCACCTGAACCGGCAGCAAATAG

Table S7. Abbreviation list for the full text

Original word	Abbreviation
Exodiol dioxygenase	EDO
Amino acid network	AAN
Cross-correlation amino acid network	CCAAN
Thermal Shift Assay	TSA
Dynamic stability centers of local structure	DSCLS
Molecular dynamics	MD
Normalized dynamic cross-correlation	NDCC
Nonlinear mutual information	NIML
wild-type	WT

The amino acid sequences of selected proteins in the paper

>MT-2
MNKGVMRPGHVQLRVLDMSKALEHYVELLGLIEMDRDDQGRVYLKAWTEVD
KFSLVLREADEPGMDFMGFKVVDEDALRQLERDLMAYGCAVEQLPAGELNSCG
RRVRFQAPSGHHFELYADKEYTGKWGLNDVNPEAWPRDLKGMAAVRFDHAL
MYGDELPATYDLFTKVLGFYLAEQVLDENGTRVAQFLSLSTKAHDVAFIHHPEK
GRLHHVSFHLETWEDLLRAADLISMTDTSIDIGPTRHGLTHGKTIYFFDPSGNRNE
VFCGGDYNYPDHKPVTWTTDQLGKAIFYHDRILNERFMTVLT

>AAC
MALVTGIGYIGIGVSDLPAWEEFAETIGFQIRERGEDGTLYLRMDKAHHRVAVHP
TGEDDLTYVGWQVADENGFDELERTLRAAGVPVEMAGEDDAELRGVARLMRF
EDPSGIKSEAYYGLVSEPEVPYVSPYAVDFVTEDQGFGHIVVMVDDYDETMRFY
REVLGLQTSDLVKVGAGGVQTRMAFMRCNPRQHSLAFWAGDSTTRLNHFMLQ
TQTLDQTGMTLDRCFHGGIPATNLGRHVNDYAVSFYITTPSGFMIEYGWGVREV
VSDYPVDKYRSVSIWGHRNLDGIHYTQALPPEAAESPAEQHLVEEPVAAAV

>R04
MTDIRGLGYLRIQTTDIARWRELVVDGLGMAIGTGPEPDGLYLRVDERRARLIVL
PGEVDKALAVGWEVRDEFALRSVREAVEKAGIAVEVLSEEESTYRDAEQVIAFD
DPGGTRTEVFFGPVLDHSPLVTPFAGVFHTGEEGLGHVVLPTAAFAESYEFYTEV
LGFLPRGATRLGGLSAPPPVRRVRFLGVNRRHHSLALCPAPPTAEPGLVHLMLEV
ETLDAVGQALYRVNKLGFSISSTLGRHTNDKMVSFYVRAPGGWDLEFGTESML
VDETFYTAEEITADSYWGHDWSGSEPLAAFSPPVG

>A2
MGEGIMRLGFVSVNVTDLEAARKHYVEVMGMQMTDRTENEIYLKGWDEYDHH
SIVLRQSNRAGLDKMAFKVHTYEDMEQLEKQLQQYGASVQRVSKGENHKVGE
GLRFRLPSGHTMELFVEMEYKGKALPQVNPAPWPEGLIGVGAPRIDHLLITAERP
HETVDFLMKALNFYMSEKVVENERSETPIAAWLFRSYTPHDIAIIPGKDEKLHHF

AFWLDEFNELRKAGDVFSKHDVPIDVGIERHGITRGQTIYYFDPSGNRNEVFTGG
YIAYPDMPVVKWTVDQLARGIFYFNHRQEWIEGFTGVTT

>L1
MNFHKEPATYVGHVHLLVSDLERSQQFYEKKLGLQVLNKKENVVAFTADGNTP
LVIIEHEENAQPKRPRTTGLYHFALLLPNRRELAKVLIHLVQSGYPLQGASDHQFS
EAVYLADPEGNGIELYADRSPEIWAWQNGELPFVSDPLDTDSLLKESENEPWTGF
PSDTVMGHIHLHVSNLQKAKEFYCDGLGFEVTVPFRHQALFVASNKYHHHIGLN
TWQGEGAPAPAANSLGMKEYSIIYPTEAERTRVLEQLKKINAPVSEEEGDVRTTD
PAGNRILLLV

>JF-8
MTAEIAKFGHIALITPNLEKSVWFFRDIVGLEEVDRQGDTIFLRAWGDWEHHTLS
LTPGNRARVDHIAWRTKRPEDVETFAEQLKAKGTEVQWIEPGEEKGQGKAIRFR
LPNGYPFEIYYDVEKPKAPEGKKSRLKNNVYRPSYGIAPRRIDHVNVWTTNPSEI
HQWLKDNMGFKMREYIRLNNGFVAGGWMSVTPLVHDIGVMVDPKGQPNRLH
HFAYYLDNVTDILRAADILREHDITIEMGGPGRHGISQAFFLYVKDPGSGHRLELF
SGGYLIFDPDWEPIEWQEHELQEGLIWYGPEMKPGGPMDDTTEC

>AAB
MALTGVLRPGYVQLRVLDLDEAIQHYRDRIGLNLVSVEGGRAFFQAFDEFDRHS
IILREADSAGLDRMAFKVARDADLDHFAERLLDIGVHVDVIAAGEDPGVGRKIRF
NTPTSHVFDLYAEMELSESGPAVRNPDVWIAEPRGMRATRFDHCALNGVDISAS
AKIFVEALDFSVAEELVDETSGVRMGIFLSCSNKAHDVAFLGYPEDGRIHHTSFFL
DSWHDVGHAADIISRYDISLDIGPTRHGITRGQTIYFFDPSGNRNETFSGGYTYYP
DNPRRMWQAENAGKAIFYYEKALNDRFMTVNT

>YAA
MKKGVMRPGHVQIRVLDMDEAVKHYKDLLGLIEMDRDDQGRVYLKAWTEVD
KFSVVLREADEPGMDFMGFKCLSEEVVDQLRGELAAFGCEIEDVPEDELKDCGR
RVRFIAPTGHSFEIYATKKQTGKWGVGNHNPEAWPRGLEGMKATRFDHClLYGP

NLDETLNLFRDVLGFDLAEQVMAPDGKRVSQFLTVSTKAHDIAFIHHEEPGKFH
HASFFLETWQDVLKAADLLSMTDTSIDIGPTRHGLTHGQTIYFFDPSGNRNEVFA
GGDYHYPDHEPVTWDAEELGKAIFYHDRVLNERFMTVLT

>C23O1
MKKGVMRPGHVQIRVLDMDEAVKHYTDLMGLIETDRDDQGRVYLKAWTEVD
KFSVVLVEADEPGCDFMGFKVVDEAALVQLEKDLIAHGLEVEQIPEGELKDCGR
RIRFTVPSGHAFELYADKLYTGKWGVTEVNPEAWPRGLSGMKAVRFDHClFYGP
ELAAVYDIFVNVLGFHLAEQVLDPEGTRIAQFLTVSMKEHDIAFIHHEEKGKFHH
ASFFLETWEDVLRAADLISMTDTSIDIGPTRHGLTHGKTIYFFDPSGNRNEVFCGG
DYTYPDHKPVTWQAEQLGKAIFYHDRVLNERFLTVLT

>APD
MIRSMAYLGLVTPAVAEWRNFGSEILGLQLIEDGEAGAARFRMDEADCRLWVH
PGEKNDIGYIGWQLTGEKEARELGDVIAAAGPVITRATPEEAAERCVAGYYWFID
PVGFRHELSWGQYVTPNSFQPGWPMSGFKTGEQGLGHIVLLVPDLPVADKFYRE
VMGFHQSDCIRDGSRALHFYHCNGRHHSLAIGSPGPGVRGAHHIMLEVNSIHDV
GKAVDRCELLDVPVSKSIGCHTNDRMVSTYIFSPSVLRVEYGFGGVEIDDLWEPK
TYSRTSIWGHKELRPDLPPAMIIEN

>1012
MGKRRHWIERLAHVELLTPKPEESLVFFRDLLGLEVTAQKGDSVYLRGWGDWF
HHTVKLTAAKEAGVGHIGWRTESPEDLEKAARFLEGKGLGVGWAEEGDLGQG
QTYRFRLPDGHQGELFFEVERYSPPSEKKPLLKNRPQKRPYRGADVRRIDHVNLY
ASDPTPSRELFQELGFKWHEGLWVKNQVEMAAWMAVSNLSHDLAFMRDPTGA
KGRLNHLAFWVDTEAEVIRAAELLREAGVFIEYGPGRHGISEAFFLYVYEPGGNR
IEVYSGGYLNFDSDWGPILWDFDELAERALATAVVGGPPPESMFAYGTPPVMLP
QS

>1028

MILRLGYAELYVTDLERARAFYVEVLGFIEAERTENRLYLRGVEEFDRYSLILTR
RDTPGLGHFGLRVASPEALSTLEKLHAALGVPLRRVPEGTLPGMGEALWVREPN
GHPVAFYHEMDQVHSFSIASSEDALPMRRTHLFRGIPPLRIDHMNLRVADVDAA
LQYWRDRLAFSVSEYVVKDGATFAAWTRRAPGTHDVALVRAKGPALHHVAYL
VQGPQEIIRTADLLADAGYQASIEFGPGRHGLSNALFLYIRDPDGNRIEIYANDYP
RDLDRPPIRWTWEEYDRRGRLWWGPEYPARFLETQPVNDRWPG

>1371
MGDIIRTARAILGVTDLERSRRFYEDALGFVLTERDDDHLYFRGYEEHVHHSLVL
KKAEVPYVEAIGFKVRSEADLDALAERFQREGRKIRWLEKGTQRAVGRALRVED
PLGVVVEYFAEMEKAERLLQRYELYRGARMQRIDHFNCLVPDVQKAYDYYTGS
LGFACSEYTATKDDRLWAAWLHRKPNVHDIAFMNGEGPRLHHIGFWVTDPMSII
HACDILASLGYAGAIERGPGRHGLSNAFFLYLRDPDGHRIELYTGDYLTSDPDFE
PIRWDVDDPRRQTFWGHAAPDVWFSEASRFKALNGDGLVPLKPPTLEKLKPTFV
L

>Monalin
GEWEIIDIGPFTQNLGKFAVDEENKIGQYGRLTFNKVIRPCMKKTIYENEGFREIK
GYEYQLYVYASDKLFRADISEDYKTRGRKLLRFNGPVPPP

>MnalinMU
NWEIIDIGPFTQNLGKFAVDEANKIGQYGRLTFNKVIRPCMKKTIYENEGFREIKG
YEYQLYVYASDKLFRADISEDYKTRGRKLLRFNGPVPPP

>caPETaseWT
DNPYQRGPDPTNASIEAATGPFAVGTQPIVGASGFGGGQIYYPTDTSQTYGAVVI
VPGFISVWAQLNWLGPRLASQGFVVIGIETSVITDLPDPRGDQALAALDWATTRS
PVASRIDRTRLAAAGWSMGGGGLRRAALQRPSLKAIVGMAPWNGERNWSAVT
VPTLFFGGSSDAVASPNDHAKPFYNSITRAEKDYIELRNADHFFPTSANTTMAKY
FISWLKRWVDNDTRYTQFLCPGPSTGLFAPVSASMNTCPF

>caPETase^{M9}

DNPYQRGPDPTNASIEAATGPFAVGTQPIVGASGFGGGQIYYPTDTSQTYGAVVI
VPGFISVWAQLAWLGPRLASQGFVVIGIETSTITDLPDPRGDQALAALDWATTRS
PVRSRIDRTRLAAAGWSMGGGGLRRAACQRPSLKAIVGMAPWNTEKNWSCVT
VPTLFFGGSSDAVASPNDHAKPFYNSITRAEKDYIELCNADHFFPTSANTTMAKY
FISWLKRWVDNDTRYTQFLCPGPSTGLFAPVCASMNTCPF

>xp-EctC

MIVRTTTEITDTDRDITSEDGNWRSKRIVLGGDRVGFSFHETTIKAGSVNEFHYAN
HVEAVWLVEGTGKLIDLDNNKEYDLAPGSMYLLNGHERHRVEPDTQMRMLCV
FNPPVTGREVHDENGVYPLVE

>N12-EctC

MIVRRLEEIIGTERDVQAKTWNSRRLLLKQDGFGYSLHDTRIFAGTETTMWYKH
HIEAVYCVEGEGELVDHETGAVHAIRPGTLYVLDRHDRHTLRAKTELRLICVFTP
ALTGQEVHDASGAYPPPEEAEA
