# Supplementary material for: A Structural Landscape Depiction of Dynamic Stability Centers of Local Structure in Protein Thermostability Engineering
Source: Research (Wash D C). 2026 Jan 5;9:1054. doi: 10.34133/research.1054 (PMC12766707; doi:10.34133/research.1054)
Supplement: Supplementary 1 — The amino acid sequences of selected proteins in the paper Figs. S1 to S35 Tables S1 to S7 [file research.1054.f1.zip › aeb6874_Suppl. Other File Type_seq1_v1.zip!/Source code.docx]

**ions.mdp**

; ions.mdp - used as input into grompp to generate ions.tpr

; Parameters describing what to do, when to stop and what to save

integrator = steep	; Algorithm (steep = steepest descent minimization)

emtol	= 1000.0	; Stop minimization when the maximum force < 1000.0 kJ/mol/nm

emstep	= 0.01	; Energy step size

nsteps	= 50000	; Maximum number of (minimization) steps to perform

; Parameters describing how to find the neighbors of each atom and how to calculate the interactions

nstlist	= 1	; Frequency to update the neighbor list and long range forces

cutoff-scheme	= Verlet

ns_type	= grid	; Method to determine neighbor list (simple, grid)

rlist	= 1.0	; cut-off for making neighbor list (short range forces)

coulombtype	= PME	; Treatment of long range electrostatic interactions

rcoulomb	= 1.0	; Short-range electrostatic cut-off

rvdw	= 1.0	; Short-range Van der Waals cut-off

pbc	= xyz	; Periodic Boundary Conditions (yes/no)

**em1.mdp**

; minim.mdp - used as input into grompp to generate em.tpr

integrator = steep	; Algorithm (steep = steepest descent minimization; cg)

emtol	= 1000.0	; Stop minimization when the maximum force < 1000.0 kJ/mol/nm

emstep	= 0.01	; Energy step size

nsteps	= 5000	; Maximum number of (minimization) steps to perform

; Parameters describing how to find the neighbors of each atom and how to calculate the interactions

nstlist	= 1	; Frequency to update the neighbor list and long range forces

cutoff-scheme	= Verlet

ns_type	= grid	; Method to determine neighbor list (simple, grid)

rlist	= 1.0	; cut-off for making neighbor list (short range forces)

coulombtype	= PME	; Treatment of long range electrostatic interactions

rcoulomb	= 1.0	; Short-range electrostatic cut-off

rvdw	= 1.0	; Short-range Van der Waals cut-off

pbc	= xyz	; Periodic Boundary Conditions (yes/no)

**em2.mdp**

; minim.mdp - used as input into grompp to generate em.tpr

integrator = cg	; Algorithm (steep = steepest descent minimization; cg)

emtol	= 1000.0	; Stop minimization when the maximum force < 1000.0 kJ/mol/nm

emstep	= 0.01	; Energy step size

nsteps	= 1000	; Maximum number of (minimization) steps to perform

; Parameters describing how to find the neighbors of each atom and how to calculate the interactions

nstlist	= 1	; Frequency to update the neighbor list and long range forces

cutoff-scheme	= Verlet

ns_type	= grid	; Method to determine neighbor list (simple, grid)

rlist	= 1.0	; cut-off for making neighbor list (short range forces)

coulombtype	= PME	; Treatment of long range electrostatic interactions

rcoulomb	= 1.0	; Short-range electrostatic cut-off

rvdw	= 1.0	; Short-range Van der Waals cut-off

pbc	= xyz	; Periodic Boundary Conditions (yes/no)

**nvt.mdp**

title	= OPLS Lysozyme NVT equilibration

define	= -DPOSRES ; position restrain the protein

; Run parameters

integrator = md	; leap-frog integrator

nsteps	= 1000000	; 2 * 50000 = 100 ps

dt	= 0.002	; 2 fs

; Output control

nstxout	= 500	; save coordinates every 1.0 ps

nstvout	= 500	; save velocities every 1.0 ps

nstenergy	= 500	; save energies every 1.0 ps

nstlog	= 500	; update log file every 1.0 ps

; Bond parameters

continuation	= no	; first dynamics run

constraint_algorithm	= lincs	; holonomic constraints

constraints	= h-bonds	; all bonds (even heavy atom-H bonds) constrained

lincs_iter	= 1	; accuracy of LINCS

lincs_order	= 4	; also related to accuracy

; Neighborsearching

cutoff-scheme	= Verlet

ns_type	= grid	; search neighboring grid cells

nstlist	= 10	; 20 fs, largely irrelevant with Verlet

rcoulomb	= 1.0	; short-range electrostatic cutoff (in nm)

rvdw	= 1.0	; short-range van der Waals cutoff (in nm)

; Electrostatics

coulombtype	= PME	; Particle Mesh Ewald for long-range electrostatics

pme_order	= 4	; cubic interpolation

fourierspacing = 0.16 ; grid spacing for FFT

; Temperature coupling is on

tcoupl	= V-rescale	; modified Berendsen thermostat

tc-grps	= system	; one coupling groups - more accurate

tau_t	= 0.1	; time constant, in ps

ref_t	= 300	; reference temperature, one for each group, in K

; Pressure coupling is off

pcoupl	= no	; no pressure coupling in NVT

; Periodic boundary conditions

pbc	= xyz	; 3-D PBC

; Dispersion correction

DispCorr	= EnerPres ; account for cut-off vdW scheme

; Velocity generation

gen_vel	= yes	; assign velocities from Maxwell distribution

gen_temp	= 300	; temperature for Maxwell distribution

gen_seed	= -1	; generate a random seed

**npt.mdp**

title	= OPLS Lysozyme NPT equilibration

define	= -DPOSRES ; position restrain the protein

; Run parameters

integrator = md	; leap-frog integrator

nsteps	= 1000000	; 50000 = 100 ps

dt	= 0.002	; 2 fs

; Output control

nstxout	= 500	; save coordinates every 1.0 ps

nstvout	= 500	; save velocities every 1.0 ps

nstenergy	= 500	; save energies every 1.0 ps

nstlog	= 500	; update log file every 1.0 ps

; Bond parameters

continuation	= yes	; Restarting after NVT

constraint_algorithm	= lincs	; holonomic constraints

constraints	= h-bonds	; all bonds (even heavy atom-H bonds) constrained

lincs_iter	= 1	; accuracy of LINCS

lincs_order	= 4	; also related to accuracy

; Neighborsearching

cutoff-scheme	= Verlet

ns_type	= grid	; search neighboring grid cells

nstlist	= 10	; 20 fs, largely irrelevant with Verlet scheme

rcoulomb	= 1.0	; short-range electrostatic cutoff (in nm)

rvdw	= 1.0	; short-range van der Waals cutoff (in nm)

; Electrostatics

coulombtype	= PME	; Particle Mesh Ewald for long-range electrostatics

pme_order	= 4	; cubic interpolation

fourierspacing = 0.16	; grid spacing for FFT

; Temperature coupling is on

tcoupl	= V-rescale	; modified Berendsen thermostat

tc-grps	= Protein Non-Protein	; two coupling groups - more accurate

tau_t	= 0.1	0.1	; time constant, in ps

ref_t	= 300	300	; reference temperature, one for each group, in K

; Pressure coupling is on

pcoupl	= berendsen	; Pressure coupling on in NPT

pcoupltype	= isotropic	; uniform scaling of box vectors

tau_p	= 2.0	; time constant, in ps

ref_p	= 1.0	; reference pressure, in bar

compressibility	= 4.5e-5	; isothermal compressibility of water, bar^-1

refcoord_scaling	= com

; Periodic boundary conditions

pbc	= xyz	; 3-D PBC

; Dispersion correction

DispCorr	= EnerPres ; account for cut-off vdW scheme

; Velocity generation

gen_vel	= no	; Velocity generation is off

**md.mdp**

title	= OPLS Lysozyme MD simulation

; Run parameters

integrator = md	; leap-frog integrator

nsteps	= 100000000 ; 2 * 500000 = 1000 ps (1 ns)

dt	= 0.002	; 2 fs

; Output control

nstxout	= 15000	; save coordinates every 30.0 ps

nstvout	= 15000	; save velocities every 30.0 ps

nstenergy	= 15000	; save energies every 30.0 ps

nstlog	= 15000	; update log file every 30.0 ps

nstxout-compressed = 15000	; save compressed coordinates every 10.0 ps

; nstxout-compressed replaces nstxtcout

compressed-x-grps	= System	; replaces xtc-grps

; Bond parameters

continuation	= yes	; Restarting after NPT

constraint_algorithm	= lincs	; holonomic constraints

constraints	= h-bonds	; all bonds (even heavy atom-H bonds) constrained

lincs_iter	= 1	; accuracy of LINCS

lincs_order	= 4	; also related to accuracy

; Neighborsearching

cutoff-scheme	= Verlet

ns_type	= grid	; search neighboring grid cells

nstlist	= 10	; 20 fs, largely irrelevant with Verlet scheme

rcoulomb	= 1.0	; short-range electrostatic cutoff (in nm)

rvdw	= 1.0	; short-range van der Waals cutoff (in nm)

; Electrostatics

coulombtype	= PME	; Particle Mesh Ewald for long-range electrostatics

pme_order	= 4	; cubic interpolation

fourierspacing = 0.16	; grid spacing for FFT

; Temperature coupling is on

tcoupl	= V-rescale	; modified Berendsen thermostat

tc-grps	= Protein Non-Protein	; two coupling groups - more accurate

tau_t	= 0.1	0.1	; time constant, in ps

ref_t	= 300	300	; reference temperature, one for each group, in K

; Pressure coupling is on

pcoupl	= berendsen	; Pressure coupling on in NPT

pcoupltype	= isotropic	; uniform scaling of box vectors

tau_p	= 2.0	; time constant, in ps

ref_p	= 1.0	; reference pressure, in bar

compressibility	= 4.5e-5	; isothermal compressibility of water, bar^-1

; Periodic boundary conditions

pbc	= xyz	; 3-D PBC

; Dispersion correction

DispCorr	= EnerPres ; account for cut-off vdW scheme

; Velocity generation

gen_vel	= no	; Velocity generation is off

**gmx-running.sh**

#!/bin/bash

pdb_file=$(ls *.pdb | head -n 1)

if [ -z "$pdb_file" ]; then

echo "未找到 PDB 文件。请确保当前目录中有 .pdb 文件。"

exit 1

fi

echo "gmx"

(echo "5"; echo "5"; echo "q") | gmx pdb2gmx -f "$pdb_file" -o oneMT2_processed.gro -p topol.top -ignh

gmx editconf -f oneMT2_processed.gro -o oneMT2_newbox.gro -c -d 1.5 -bt cubic

gmx solvate -cp oneMT2_newbox.gro -cs spc216.gro -o oneMT2_solv.gro -p topol.top

gmx grompp -f ions.mdp -c oneMT2_solv.gro -p topol.top -o ions.tpr -maxwarn 1

(echo "13"; echo "q") | gmx genion -s ions.tpr -o solv_ions.gro -p topol.top -pname NA -nname CL -neutral

gmx grompp -f em1.mdp -c solv_ions.gro -p topol.top -o em.tpr -maxwarn 7

gmx mdrun -v -deffnm em

gmx grompp -f em2.mdp -c em.gro -p topol.top -o em.tpr -maxwarn 7

gmx mdrun -v -deffnm em

(echo "13"; echo "q") | gmx make_ndx -f em.gro -o index.ndx

gmx grompp -f nvt.mdp -c em.gro -r em.gro -p topol.top -n index.ndx -o nvt.tpr -maxwarn 6

gmx mdrun -v -deffnm nvt

gmx grompp -f npt.mdp -c nvt.gro -r nvt.gro -t nvt.cpt -p topol.top -n index.ndx -o npt.tpr -maxwarn 6

gmx mdrun -v -deffnm npt

**gromacs.slurm**

#!/bin/bash

#SBATCH -J gromacs

#SBATCH -N 1

#SBATCH --ntasks-per-node=1

#SBATCH --cpus-per-task=6

#SBATCH --gres=gpu:1

#SBATCH -p dgx2

module purge

module load gromacs/2024.1-gcc-12.3.0-cuda

gmx_mpi grompp -f md.mdp -c npt.gro -r npt.gro -t npt.cpt -p topol.top -n index.ndx -o md_0_1.tpr -maxwarn 6

gmx_mpi mdrun -v -deffnm md_0_1

**cluster-emnerg.py**

import MDAnalysis as mda

from MDAnalysis.analysis import pca

import numpy as np

import matplotlib.pyplot as plt

from matplotlib import rcParams

from scipy.stats import gaussian_kde

from scipy.cluster.hierarchy import linkage, dendrogram, fcluster

from scipy.spatial.distance import cdist

import seaborn as sns

rcParams['font.family'] = 'DejaVu Sans'

u = mda.Universe('/home/dragon/data2/QX/MT2/output.gro', '/home/dragon/data2/QX/MT2/md_0_1_processed.xtc')

pca_analysis = pca.PCA(u, select='protein and backbone')

pca_results = pca_analysis.run()

principal_components = pca_results.results.p_components

pc1_values = principal_components[:, 0]

pc2_values = principal_components[:, 1]

kde = gaussian_kde([pc1_values, pc2_values])

xgrid, ygrid = np.mgrid[min(pc1_values):max(pc1_values):100j, min(pc2_values):max(pc2_values):100j]

z = kde(np.vstack([xgrid.ravel(), ygrid.ravel()]))

free_energy = -np.log(z).reshape(xgrid.shape)

data = np.vstack((pc1_values, pc2_values)).T

linkage_matrix = linkage(data, method='ward')

plt.figure(figsize=(10, 6))

dendrogram(linkage_matrix, labels=np.arange(len(pc1_values)), leaf_rotation=90)

plt.title('Hierarchical Clustering Dendrogram')

plt.xlabel('Samples')

plt.ylabel('Distance')

plt.savefig('hierarchical_clustering_dendrogram.pdf', format='pdf')

plt.close()

num_clusters = 5

cluster_labels = fcluster(linkage_matrix, t=num_clusters, criterion='maxclust')

cluster_centers = []

representative_indices = []

for cluster_id in range(1, num_clusters + 1):

cluster_points = data[cluster_labels == cluster_id]

cluster_indices = np.where(cluster_labels == cluster_id)[0]

center = cluster_points.mean(axis=0)

cluster_centers.append(center)

distances = cdist([center], cluster_points)[0]

representative_index = cluster_indices[np.argmin(distances)]

representative_indices.append(representative_index)

for i, index in enumerate(representative_indices):

u.trajectory[index]

output_filename = f'cluster_{i + 1}_center.pdb'

with mda.Writer(output_filename, multiframe=False) as writer:

writer.write(u.atoms)

print(f"Cluster {i + 1} center saved to {output_filename}")

plt.figure(figsize=(10, 8))

contour = plt.contourf(xgrid, ygrid, free_energy, levels=50, cmap='viridis', alpha=0.7)

plt.colorbar(contour, label='Free Energy (kcal/mol)')

plt.scatter(pc1_values, pc2_values, s=10, alpha=0.6, color='black')

plt.xlabel('Principal Component 1 (PC1)')

plt.ylabel('Principal Component 2 (PC2)')

plt.title('Free Energy Landscape and Clustering Analysis of Protein Conformations')

plt.savefig('protein_conformations_hierarchical_clustering.pdf', format='pdf')

plt.close()

**Fnet.py**

import networkx as nx

import numpy as np

import community as community_louvain

def calculate_modularity(G, components):

m = G.number_of_edges()

Q = 0.0

for community in components:

subgraph = G.subgraph(community)

l_c = subgraph.number_of_edges()

d_c = sum(dict(G.degree(community)).values())

Q += (l_c / m) - (d_c / (2 * m)) ** 2

return Q

def louvain_community_detection(G):

partition = community_louvain.best_partition(G, weight='weight')

communities = {}

for node, community_id in partition.items():

if community_id not in communities:

communities[community_id] = []

communities[community_id].append(node)

return list(communities.values())

def get_leaders_and_followers(G, communities):

leaders = []

followers = []

for community in communities:

community_subgraph = G.subgraph(community)

betweenness = nx.betweenness_centrality(community_subgraph)

leader = max(betweenness, key=betweenness.get)

leaders.append(leader)

for node in community:

neighbors_in_community = sum(1 for neighbor in G.neighbors(node) if neighbor in community)

if neighbors_in_community == len(list(G.neighbors(node))):

followers.append(node)

return leaders, followers

def clique_percolation_algorithm(G, k):

cliques = list(nx.find_cliques(G))

communities = []

visited = set()

for clique in cliques:

if len(clique) >= k:

clique_set = set(clique)

if not visited.intersection(clique_set):

community = clique_set.copy()

for node in clique:

for neighbor in G.neighbors(node):

if neighbor not in visited:

community.add(neighbor)

communities.append(community)

visited.update(community)

return communities

def create_graph_from_matrix(matrix_file, is_ndcc=False, threshold=0.45):

matrix = np.loadtxt(matrix_file)

if is_ndcc:

matrix = (matrix + 1) / 2

matrix[matrix < threshold] = 0

G = nx.Graph()

for i in range(matrix.shape[0]):

for j in range(i + 1, matrix.shape[1]):

if matrix[i, j] > 0:

G.add_edge(i, j, weight=matrix[i, j])

G.remove_nodes_from(list(nx.isolates(G)))

return G

def find_inter_community_nodes(G, communities):

node_to_community = {}

for i, community in enumerate(communities):

for node in community:

node_to_community[node] = i

inter_community_nodes = {}

for node in G.nodes:

neighbor_communities = set(node_to_community.get(neighbor) for neighbor in G.neighbors(node))

current_community = node_to_community.get(node, -1)

cross_community_count = len(neighbor_communities - {current_community})

if cross_community_count > 0:

inter_community_nodes[node] = cross_community_count

sorted_nodes = sorted(inter_community_nodes.items(), key=lambda x: x[1], reverse=True)

return sorted_nodes

nlmi_G = create_graph_from_matrix('nlmi-6lu7-md.dat', is_ndcc=False, threshold=0.45)

ndcc_G = create_graph_from_matrix('ndcc-6lu7-md.dat', is_ndcc=True, threshold=0.7225)

communities_nlmi_louvain = louvain_community_detection(nlmi_G)

communities_ndcc_louvain = louvain_community_detection(ndcc_G)

finer_communities_nlmi_louvain = []

for community in communities_nlmi_louvain:

subgraph = nlmi_G.subgraph(community)

finer_communities_nlmi_louvain.extend(clique_percolation_algorithm(subgraph, k=4))

finer_communities_ndcc_louvain = []

for community in communities_ndcc_louvain:

subgraph = ndcc_G.subgraph(community)

finer_communities_ndcc_louvain.extend(clique_percolation_algorithm(subgraph, k=4))

leaders_nlmi, followers_nlmi = get_leaders_and_followers(nlmi_G, finer_communities_nlmi_louvain)

leaders_ndcc, followers_ndcc = get_leaders_and_followers(ndcc_G, finer_communities_ndcc_louvain)

print("Louvain Communities (NLMI):")

print(communities_nlmi_louvain)

print("Finer Communities (NLMI) after CPA:")

print(finer_communities_nlmi_louvain)

print("Louvain Communities (NDCC):")

print(communities_ndcc_louvain)

print("Finer Communities (NDCC) after CPA:")

print(finer_communities_ndcc_louvain)

print("\nLeaders (NLMI):")

print(leaders_nlmi)

print("\nFollowers (NLMI):")

print(followers_nlmi)

print("\nLeaders (NDCC):")

print(leaders_ndcc)

print("\nFollowers (NDCC):")

print(followers_ndcc)

inter_community_nodes_nlmi = find_inter_community_nodes(nlmi_G, finer_communities_nlmi_louvain)

print("\n关键跨社区节点（NLMI）：")

for node, connections in inter_community_nodes_nlmi:

print(f"节点 {node} 连接了 {connections} 个不同社区")

inter_community_nodes_ndcc = find_inter_community_nodes(ndcc_G, finer_communities_ndcc_louvain)

print("\n关键跨社区节点（NDCC）：")

for node, connections in inter_community_nodes_ndcc:

print(f"节点 {node} 连接了 {connections} 个不同社区")

**Lnet-2.py**

import networkx as nx

import numpy as np

import community as community_louvain

def calculate_modularity(G, components):

m = G.number_of_edges()

Q = 0.0

for community in components:

subgraph = G.subgraph(community)

l_c = subgraph.number_of_edges()

d_c = sum(dict(G.degree(community)).values())

Q += (l_c / m) - (d_c / (2 * m)) ** 2

return Q

def louvain_community_detection(G):

partition = community_louvain.best_partition(G, weight='weight')

communities = {}

for node, community_id in partition.items():

if community_id not in communities:

communities[community_id] = []

communities[community_id].append(node)

return list(communities.values())

def get_leaders_and_followers(G, communities):

leaders = []

followers = []

for community in communities:

community_subgraph = G.subgraph(community)

betweenness = nx.betweenness_centrality(community_subgraph)

leader = max(betweenness, key=betweenness.get)

leaders.append(leader)

for node in community:

neighbors_in_community = sum(1 for neighbor in G.neighbors(node) if neighbor in community)

if neighbors_in_community == len(list(G.neighbors(node))):

followers.append(node)

return leaders, followers

def clique_percolation_algorithm(G, k):

cliques = list(nx.find_cliques(G))

communities = []

visited = set()

for clique in cliques:

if len(clique) >= k:

clique_set = set(clique)

if not visited.intersection(clique_set):

community = clique_set.copy()

for node in clique:

for neighbor in G.neighbors(node):

if neighbor not in visited:

community.add(neighbor)

communities.append(community)

visited.update(community)

return communities

def create_graph_from_matrix(matrix_file, is_ndcc=False, threshold=0.45):

matrix = np.loadtxt(matrix_file)

if is_ndcc:

matrix = (matrix + 1) / 2

matrix[matrix < threshold] = 0

G = nx.Graph()

for i in range(matrix.shape[0]):

for j in range(i + 1, matrix.shape[1]):

if matrix[i, j] > 0:

G.add_edge(i, j, weight=matrix[i, j])

G.remove_nodes_from(list(nx.isolates(G)))

return G

def find_inter_community_nodes(G, communities):

node_to_community = {}

for i, community in enumerate(communities):

for node in community:

node_to_community[node] = i

inter_community_nodes = {}

for node in G.nodes:

neighbor_communities = set(node_to_community.get(neighbor) for neighbor in G.neighbors(node))

current_community = node_to_community.get(node, -1)

cross_community_count = len(neighbor_communities - {current_community})

if cross_community_count > 0:

inter_community_nodes[node] = cross_community_count

sorted_nodes = sorted(inter_community_nodes.items(), key=lambda x: x[1], reverse=True)

return sorted_nodes

nlmi_G = create_graph_from_matrix('nlmi-6lu7-md.dat', is_ndcc=False, threshold=0.45)

ndcc_G = create_graph_from_matrix('ndcc-6lu7-md.dat', is_ndcc=True, threshold=0.7225)

communities_nlmi_louvain = louvain_community_detection(nlmi_G)

communities_ndcc_louvain = louvain_community_detection(ndcc_G)

finer_communities_nlmi_louvain = []

for community in communities_nlmi_louvain:

subgraph = nlmi_G.subgraph(community)

finer_communities_nlmi_louvain.extend(clique_percolation_algorithm(subgraph, k=4))

finer_communities_ndcc_louvain = []

for community in communities_ndcc_louvain:

subgraph = ndcc_G.subgraph(community)

finer_communities_ndcc_louvain.extend(clique_percolation_algorithm(subgraph, k=4))

leaders_nlmi, followers_nlmi = get_leaders_and_followers(nlmi_G, finer_communities_nlmi_louvain)

leaders_ndcc, followers_ndcc = get_leaders_and_followers(ndcc_G, finer_communities_ndcc_louvain)

print("Louvain Communities (NLMI):")

print(communities_nlmi_louvain)

print("Finer Communities (NLMI) after CPA:")

print(finer_communities_nlmi_louvain)

print("Louvain Communities (NDCC):")

print(communities_ndcc_louvain)

print("Finer Communities (NDCC) after CPA:")

print(finer_communities_ndcc_louvain)

print("\nLeaders (NLMI):")

print(leaders_nlmi)

print("\nFollowers (NLMI):")

print(followers_nlmi)

print("\nLeaders (NDCC):")

print(leaders_ndcc)

print("\nFollowers (NDCC):")

print(followers_ndcc)

inter_community_nodes_nlmi = find_inter_community_nodes(nlmi_G, finer_communities_nlmi_louvain)

print("\n关键跨社区节点（NLMI）：")

for node, connections in inter_community_nodes_nlmi:

print(f"节点 {node} 连接了 {connections} 个不同社区")

inter_community_nodes_ndcc = find_inter_community_nodes(ndcc_G, finer_communities_ndcc_louvain)

print("\n关键跨社区节点（NDCC）：")

for node, connections in inter_community_nodes_ndcc:

print(f"节点 {node} 连接了 {connections} 个不同社区")

**Three angle.py**

import numpy as np

import MDAnalysis as mda

from MDAnalysis.analysis import dihedrals

def calcMDsingleDihedralCC(topology, trajectory, startingFrame=0, endingFrame=(-1),

normalized=True, dihedralType="psi", saveMatrix=True, out_file="dihedral-cc.dat"):

debug = False

\# Create the universe (That sounds really fancy :)

universe = mda.Universe(topology, trajectory)

\# Create an atomgroup from the protein atoms selection

proteinAtoms = universe.select_atoms("protein")

print(f"@> Parsed {proteinAtoms.n_atoms} protein atoms.")

\# Set your frame window for your trajectory that you want to analyze

if endingFrame == -1:

endingFrame = universe.trajectory.n_frames

noneIndex = 0

noneList = []

\# Select dihedral type and collect valid dihedrals

if dihedralType == 'omega':

selected_dihs = []

for res in proteinAtoms.residues:

omega = res.omega_selection()

if omega is not None: # Only add valid dihedral

selected_dihs.append(omega)

else:

noneList.append(noneIndex)

noneIndex += 1

elif dihedralType == 'phi':

selected_dihs = []

for res in proteinAtoms.residues:

phi = res.phi_selection()

if phi is not None: # Only add valid dihedral

selected_dihs.append(phi)

else:

noneList.append(noneIndex)

noneIndex += 1

elif dihedralType == 'psi':

selected_dihs = []

for res in proteinAtoms.residues:

psi = res.psi_selection()

if psi is not None: # Only add valid dihedral

selected_dihs.append(psi)

else:

noneList.append(noneIndex)

noneIndex += 1

\# Make sure there's at least one dihedral

if not selected_dihs:

raise ValueError(f"No valid {dihedralType} dihedrals found in the selected protein")

\# Run dihedral analysis

dih_analysis = dihedrals.Dihedral(selected_dihs).run(start=startingFrame, stop=endingFrame)

\# Convert angles from degrees to radians, then calculate cosine

Rvector = np.cos(dih_analysis.angles * np.pi / 180)

\# Handle residues without dihedral angles (fill them with 1.0)

for missing in noneList:

Rvector = np.insert(Rvector, missing, 1.0, axis=1)

\# Number of frames in the trajectory

N_Frames = len(Rvector)

R_average = np.mean(Rvector, axis=0)

\# Handle missing residues' average values (set to 0.0)

for missing in noneList:

R_average[missing] = 0.0

print("@> Calculating cross-correlation matrix:")

N = len(R_average) # The number of dihedral angles (residues)

\# Initialize the correlation matrix

ccMatrix = np.zeros((N, N), np.double)

for k in range(0, len(Rvector)):

if k % 100 == 0:

print("@> Frame: " + str(k))

deltaR = np.subtract(Rvector[k], R_average)

ccMatrix += np.outer(deltaR, deltaR)

\# Average the correlation matrix

ccMatrix = ccMatrix / float(N_Frames)

\# Normalize if requested

if normalized:

cc_normalized = np.zeros((N, N), np.double)

for i in range(0, N):

for j in range(i, N):

cc_normalized[i][j] = ccMatrix[i][j] / ((ccMatrix[i][i] * ccMatrix[j][j]) ** 0.5)

cc_normalized[j][i] = cc_normalized[i][j]

if saveMatrix:

np.savetxt(out_file, cc_normalized, fmt='%.6f')

return cc_normalized

else:

for i in range(0, N):

for j in range(i + 1, N):

ccMatrix[j][i] = ccMatrix[i][j]

if saveMatrix:

np.savetxt(out_file, ccMatrix, fmt='%.6f')

return ccMatrix

\# Example usage

topology = "fixmt2.pdb"

trajectory = "Hnew_md_0_1.trr"

\# Calculate omega cross-correlation matrix

omegacc = calcMDsingleDihedralCC(topology, trajectory, dihedralType="omega", out_file="omega-cc.dat")

\# Calculate phi cross-correlation matrix

phicc = calcMDsingleDihedralCC(topology, trajectory, dihedralType="phi", out_file="phi-cc.dat")

\# Calculate psi cross-correlation matrix

psicc = calcMDsingleDihedralCC(topology, trajectory, dihedralType="psi", out_file="psi-cc.dat")

**netpointcheck-1.py**

import pandas as pd

import networkx as nx

import matplotlib.pyplot as plt

file_list = [

'cluster_1_center.txt',

'cluster_2_center.txt',

'cluster_3_center.txt',

'cluster_4_center.txt',

'cluster_5_center.txt'

]

sorted_unique_numbers = [15, 21, 29, 31, 32, 43, 44, 45, 46, 48, 49, 50, 56, 79, 113, 114, 123, 137, 151, 158, 168, 178, 180, 182, 183, 184, 185, 186, 189, 195, 200, 206, 207, 212, 215, 232, 234, 235, 240, 244, 245, 247, 259, 262, 263, 274, 275, 277, 280, 282, 285, 289, 290, 292, 293, 294, 296, 302, 305]

def get_residue_id(node_id):

try:

parts = node_id.split(":")

if len(parts) >= 2:

return int(parts[1])

else:

return None

except Exception as e:

print(f"错误: 无法处理NodeId: {node_id}. 错误信息: {e}")

return None

G_combined = nx.Graph()

for file_path in file_list:

try:

data = pd.read_csv(file_path, sep="\t", header=None)

data.columns = ["NodeId1", "Interaction", "NodeId2", "Distance", "Angle", "Energy", "Atom1", "Atom2", "Donor", "Positive", "Cation", "Orientation", "Model"]

except Exception as e:

print(f"错误: 读取文件 {file_path} 时发生问题. 错误信息: {e}")

continue

filtered_data = data[

data['NodeId1'].apply(lambda x: get_residue_id(x) in sorted_unique_numbers) |

data['NodeId2'].apply(lambda x: get_residue_id(x) in sorted_unique_numbers)

]

print(f"筛选前数据大小: {len(data)}")

print(f"筛选后数据大小: {len(filtered_data)}")

output_text_file = file_path.replace('.txt', '_filtered.txt')

filtered_data.to_csv(output_text_file, sep="\t", index=False)

print(f"筛选后的数据保存为: {output_text_file}")

for _, row in filtered_data.iterrows():

residue1 = get_residue_id(row['NodeId1'])

residue2 = get_residue_id(row['NodeId2'])

if residue1 is not None and residue2 is not None:

interaction = row['Interaction']

distance = row['Distance']

G_combined.add_edge(residue1, residue2, interaction=interaction, distance=distance)

plt.figure(figsize=(12, 8))

pos = nx.spring_layout(G_combined)

nx.draw(G_combined, pos, with_labels=True, node_size=500, font_size=10, font_color="white", node_color="blue", edge_color="gray")

edge_labels = {(u, v): f"{d['interaction']} ({float(d['distance']):.2f} Å)" for u, v, d in G_combined.edges(data=True)}

nx.draw_networkx_edge_labels(G_combined, pos, edge_labels=edge_labels)

output_combined_graph_file = 'combined_network.png'

plt.title("Combined Network of Residues Interactions")

plt.savefig(output_combined_graph_file)

plt.close()

print(f"合并后的网络图保存为: {output_combined_graph_file}")

unique_edges = set()

for u, v in G_combined.edges():

unique_edges.add(tuple(sorted((u, v))))

with open('combined_network_connections.txt', 'w') as f:

for u, v, data in G_combined.edges(data=True):

interaction = data.get('interaction', 'Unknown')

f.write(f"{u}\t{v}\t{interaction}\n")

print(f"去除重复连接并包含交互类型的文本文件保存为: combined_network_connections.txt")

**去活性位点后的位点检测.py**

from collections import defaultdict

def dfs(graph, node, visited, component, interactions):

visited.add(node)

component.append(node)

for neighbor, interaction in graph[node]:

if neighbor not in visited:

interactions[interaction].append((node, neighbor))

dfs(graph, neighbor, visited, component, interactions)

def partition_nodes_from_file(filename):

graph = defaultdict(list)

interactions = defaultdict(list)

with open(filename, 'r') as file:

for line in file:

if line.strip():

u, v, interaction = line.strip().split()

u, v = int(u), int(v)

graph[u].append((v, interaction))

graph[v].append((u, interaction))

components = []

visited = set()

for node in graph:

if node not in visited:

component = []

component_interactions = defaultdict(list)

dfs(graph, node, visited, component, component_interactions)

components.append((sorted(component), component_interactions))

return components

filename = 'combined_network_connections.txt'

partitioned_nodes = partition_nodes_from_file(filename)

for i, (component, component_interactions) in enumerate(partitioned_nodes):

print(f"连通分量 {i+1}: {sorted(component)}")

for interaction_type, edges in component_interactions.items():

print(f" {interaction_type}: {edges}")
